# Indigenous Australian genomes show deep structure and rich novel variation

**DOI:** 10.1038/s41586-023-06831-w

**Published:** 2023-12-13

**Authors:** Matthew Silcocks, Ashley Farlow, Azure Hermes, Georgia Tsambos, Hardip R. Patel, Sharon Huebner, Gareth Baynam, Misty R. Jenkins, Damjan Vukcevic, Simon Easteal, Stephen Leslie, The National Centre for Indigenous Genomics, Ashley Farlow, Azure Hermes, Hardip R. Patel, Sharon Huebner, Gareth Baynam, Misty R. Jenkins, Simon Easteal, Stephen Leslie

**Affiliations:** 1 National Centre for Indigenous Genomics, John Curtin School of Medical Research, Australian National University, Canberra, Australian Capital Territory Australia; 2 University of Melbourne, School of Biosciences, Parkville, Victoria Australia; 3 University of Melbourne, School of Mathematics and Statistics, Parkville, Victoria Australia; 4 Faculty of Health and Medical Sciences, Division of Paediatrics and Telethon Kids Institute, University of Western Australia, Perth, Western Australia Australia; 5 Western Australian Register of Developmental Anomalies, King Edward Memorial Hospital and Rare Care Centre, Perth Children’s Hospital, Perth, Western Australia Australia; 6 Immunology Division, The Walter and Eliza Hall Institute of Medical Research, Parkville, Victoria Australia; 7 University of Melbourne, Department of Medical Biology, Parkville, Victoria Australia; 8 John Curtin School of Medical Research, Australian National University, Canberra, Australian Capital Territory Australia

**Keywords:** Medical genomics, Rare variants, Population genetics, Evolutionary genetics

## Abstract

The Indigenous peoples of Australia have a rich linguistic and cultural history. How this relates to genetic diversity remains largely unknown because of their limited engagement with genomic studies. Here we analyse the genomes of 159 individuals from four remote Indigenous communities, including people who speak a language (Tiwi) not from the most widespread family (Pama–Nyungan). This large collection of Indigenous Australian genomes was made possible by careful community engagement and consultation. We observe exceptionally strong population structure across Australia, driven by divergence times between communities of 26,000–35,000 years ago and long-term low but stable effective population sizes. This demographic history, including early divergence from Papua New Guinean (47,000 years ago) and Eurasian groups^1^, has generated the highest proportion of previously undescribed genetic variation seen outside Africa and the most extended homozygosity compared with global samples. A substantial proportion of this variation is not observed in global reference panels or clinical datasets, and variation with predicted functional consequence is more likely to be homozygous than in other populations, with consequent implications for medical genomics^2^. Our results show that Indigenous Australians are not a single homogeneous genetic group and their genetic relationship with the peoples of New Guinea is not uniform. These patterns imply that the full breadth of Indigenous Australian genetic diversity remains uncharacterized, potentially limiting genomic medicine and equitable healthcare for Indigenous Australians.

## Abstract (web summary)

Analysis of the genomes of 159 individuals from four Indigenous communities in Australia shows a high level of genetic variation and demonstrates the need for greater representation of Indigenous Australians in reference panels and clinical databases.

## Main

The Indigenous populations of Australia remain poorly represented in sequencing panels and clinical databases. Their inclusion is warranted on the grounds of equity and their unique demographic history. Indigenous Australians probably descend from an early dispersal of humans across Asia^3^, inheriting substantial ancestry from extinct hominin groups^1,4,5^. Previous DNA studies have identified novel variation^6^ and inferred a long history of geographical regionalism in Australia^7^. An earlier whole-genome sequencing study inferred a sudden separation from Papuans 25–40 thousand years ago (ka) and divergence within Australia occurring 10–32 ka (ref. ^1^). Importantly, all 83 participants in the study were Pama–Nyungan language speakers, a language family that is widespread across Australia despite its relatively recent origin (estimated at 6 ka)^8^, possibly accounting for the lack of strong discernible structure^1^. It is estimated that another 27 language families^9^, largely restricted to the Top End and Kimberley region, are unrepresented in genomic data. Linguistic variation is often correlated with patterns of genetic variation^10^, supporting the inclusion of speakers of these languages in genomics studies.

If limited population structure remains after more representative geographical and language group sampling, a common set of genomic tools and reference panels will be sufficient to inform medical research and clinical practice. Alternatively, previously undocumented structure, due to patterns of migration, isolation and population size change, may indicate the poor suitability of such panels and support wider sampling to capture the full distribution and diversity of common and rare alleles.

Such patterns can be explored by quantifying the levels of novel and shared variation relative to other human populations and by applying population genetic models to determine structure and its causes. Both approaches require adequate sampling within communities and the inclusion of communities that capture the breadth of the underlying genetic diversity.

## The NCIG collection

The Australian National University holds more than 7,000 biospecimens collected between the 1960s and 1990s from about 40 Indigenous communities (Supplementary Note 1). A panel of leading Aboriginal and Torres Strait Islander Australians recommended the collection be placed under Indigenous-majority custodianship, leading to the establishment of the National Centre for Indigenous Genomics (NCIG) in 2016^11^. The primary role of NCIG is to engage with Indigenous communities on the existence and nature of the collection, extend and promote its use for research and ensure that research is done with appropriate personal consent and community engagement (Methods).

During recent community engagement, 159 community members provided new blood or saliva samples under modern consent and ethics protocols. This study analyses genetic data from these Indigenous Australians from four environmentally diverse regions across northern and central Australia, including tropical savannah and rainforest, remote islands and desert. (Clearly these environments will have varied over the many millennia Indigenous Australians have lived on the continent). This is a large and purposefully diverse collection of genomic data from Indigenous Australians.

The cohort includes 59 individuals from the Tiwi Islands. The Tiwi people experienced a long period of isolation from mainland Australia^12^ and speak a linguistic isolate unrelated to the Pama–Nyungan languages spoken by the other three communities involved. Included are 33 people from the community of Wurrumiyanga on Bathurst Island, 20 from Milikapiti and six from Pirlangimpi on Melville Island. This is about 3% of the current population of the islands (around 2,000). The cohort also includes 48 individuals from the community of Yarrabah on the traditional lands of the Gunggandji and Mandingalbay Yidinji. The Yarrabah Aboriginal Mission, established in 1892, was used as a settlement for displaced Indigenous people from across Queensland. In 1938, 43 different tribal groups were represented in Yarrabah^13^. The cohort contains 14 people from the Central Desert community of Titjikala, comprising of members of the Southern Arrernte, Yankunytjatjara, Luritja and Pitjantjatjara. Finally, there are 38 individuals from the community of Galiwin’ku on Elcho Island. Established in 1942, the community comprises members of 30 closely related clan groups (Yalu team Galiwin’ku, personal communication).

DNA was extracted from either blood or saliva and Illumina sequenced to high coverage (minimum 30×, median 42×; see Methods and Supplementary Note 2). Variants were called jointly and phased with 60 previously sequenced individuals from geographically adjacent populations (25 men from the highlands of Papua New Guinea (PNG) drawn from five different language groups^1^ and 35 men from 11 regions of the Bismarck Archipelago of PNG in Island Melanesia^5^).

## Genetic ancestry in the collection

**Extended Data Fig. 1:**
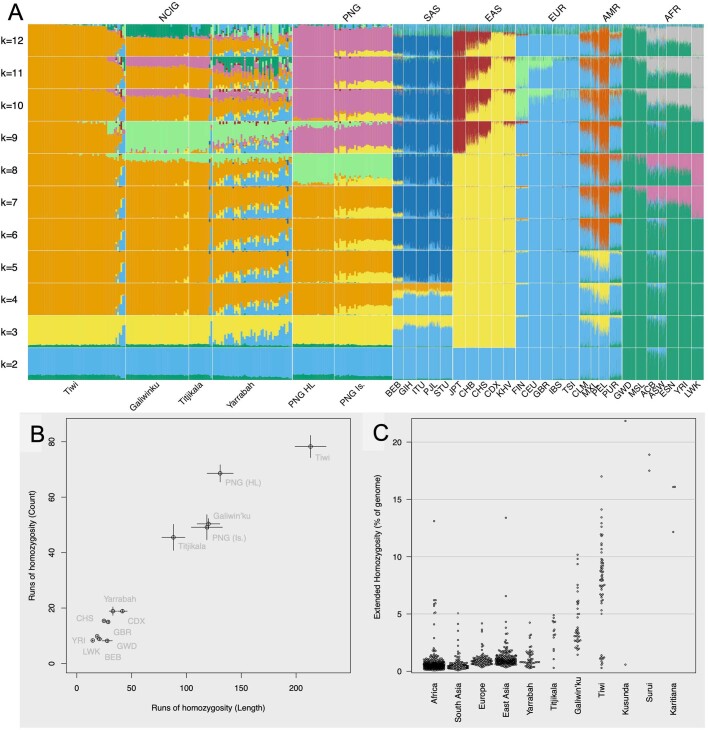
Global ancestry and homozygosity. **A**. Global ancestry proportions for NCIG, Papuan and 1000 Genomes. The software ADMIXTURE run with 159 Indigenous Australian samples, 60 Papuan and 2,600 samples from the LC 1000 Genomes. Samples shown horizontally by population (with reduced bar width for the 1000 Genomes samples) and cluster (colour) assignment proportion shown as bar height. ADMIXTURE was run assuming the sample contained from k = 2 up to k = 12 clusters (y-axis). No ancestry mask applied. Restricted to biallelic SNVs, MAF > 0.01 and LD thinned. This analysis was used to estimate non-Indigenous Australian or PNG ancestry in the NCIG and PNG samples. **B**. Runs of homozygosity (ROH) for the NCIG + PNG (unmasked) dataset and a subset of (HC) 1000 Genomes samples. Mean count versus mean sum of ROH segments greater than 1 Mb in length. Error bars are within population SEM. Note that a long-term reduction in effective population size is expected to increase both the count and total length of ROH (as seen for NCIG populations), whereas recent consanguinity generates a small number of long ROH. **C**. Per-individual ROH length (for ROH > 1 Mb) as a percentage of the total autosomal genome length (2.8 Gb). Individuals from three Indigenous populations from Nepal (Kusunda) and Brazil (Surui and Karitiana) from the SGDP were included for comparison as some exhibited extreme levels of ROH. Individuals identified as “Tiwi-outliers” were included in the Tiwi sample in this plot and are evident in panel C as a cluster of individuals with reduced ROH.

We emphasize that genetic ancestry proportions may or may not align with identity and that all communities worldwide have varying degrees of shared ancestry. Nonetheless, we seek to focus on genetic ancestry that is Indigenous Australian in origin. Thus, our cohort was combined with the 1000 Genomes Project samples^14^ (hereafter 1000 Genomes), and we applied standard algorithms to identify genomic regions with ancestry other than Indigenous ancestry (Methods and Supplementary Note 2). We find that 100 of 111 individuals from Titjikala, Galiwin’ku and Tiwi have only Indigenous ancestry (Extended Data Fig. 1a). By contrast, consistent with the history of the community, all Yarrabah individuals have an appreciable degree of European, East Asian and/or putative Melanesian ancestry (mean 41%, range 11–73%). Notably, and consistent with known sex-specific demographic patterns^1,15^, all Australian individuals have a mitochondrial lineage belonging to a previously documented Indigenous Australian haplogroup (see ‘Mitochondrial diversity’ section).

To avoid genomic regions of non-Indigenous ancestry confounding analyses, local ancestry was inferred along each haplotype on the basis of a reference panel of individuals thought to be unadmixed from Australia, PNG, Eurasia and Africa. Genomic regions were masked within an individual if one or both haplotypes were inferred to be of non-Indigenous ancestry: that is, neither Australian nor Papuan (see ‘Ancestry inference’ in Methods and Supplementary Note 2). Ten individuals from Tiwi showed patterns of polymorphism and clustering consistent with having at least one recent ancestor from an Indigenous community other than Tiwi (Supplementary Note 2). Unless otherwise stated, all analyses were performed on this ancestry-masked dataset, filtered to remove these ten Tiwi individuals and first- and second-degree relatives, leaving 89 individuals (34 Tiwi, 31 Yarrabah, 17 Galiwin’ku, 7 Titjikala).

The size of this collection, its geographical distribution and the limited non-Indigenous ancestry is notable compared with previous studies^1,16,17^. This allowed for characterization of novel and shared genetic variation at the individual and population levels and inference of the demographic forces that have generated these patterns.

## Australian variation in a global context

The suitability of current reference databases for genomics involving Indigenous Australians depends on how well they capture variation in these populations. Of the 9.9 million single-nucleotide variants (SNVs) observed across all 159 individuals after ancestry masking, 3.4 million (34%) are not present in either the 1000 Genomes^18^ or the Human Genome Diversity Project (HGDP)^19^ (Extended Data Table 1). For comparison, only 10% of SNVs observed in the analysed Papuan individuals are absent from both datasets, probably because of the Papuan samples in the HGDP. Of the variants seen in the Australian cohort, 26% are not observed in either PNG individuals or the Genome Aggregation Database (gnomAD) release 3.1 (which has 76,000 samples, including the 1000 Genomes and HGDP)^20^. This is important as rarity in gnomAD is one metric used to prioritize potentially pathogenic variants for clinical diagnostics. Out of all variants observed, 2.1 million are restricted to a single Indigenous Australian population sample. Thus, given the limitation of current sampling, between 6.3% and 8.7% of SNVs in each of these four population samples are not observed elsewhere.

**Fig. 1:**
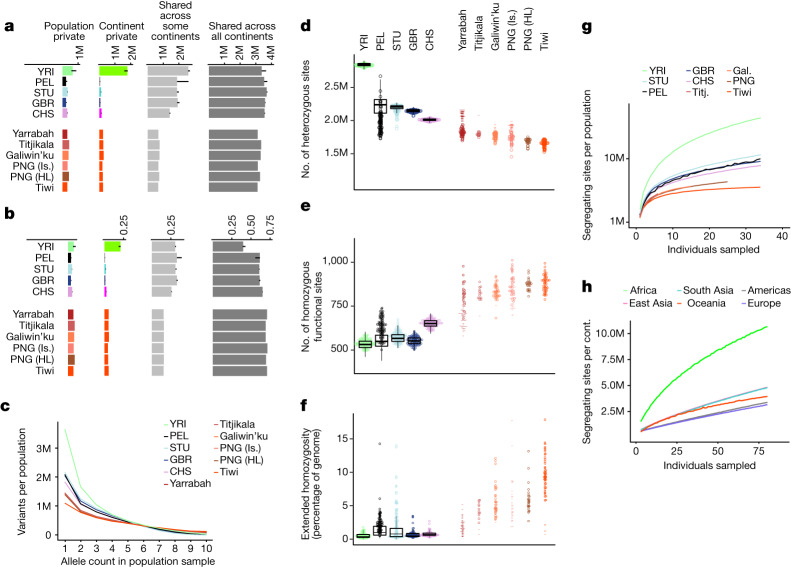
Variant characteristics across populations. **a**,**b**, Per-population count (**a**) and proportion of total variation (**b**) for biallelic SNVs across four classes of sharing for samples of five individuals per population (samples of 15 and 25 in Supplementary Fig. 1). Bars: values for single representative populations. Lines: range for other continental populations. Sharing defined relative to all 26 populations of the 1000 Genomes and the six Oceanic populations considered here. **c**, Distribution of minor allele count within each population sample (restricted to five as above). Minor allele defined by pooling the five individuals from each of the 32 populations. **d**–**f**, Per-individual count of heterozygous sites (**d**), homozygous amino acid substitutions with predicted functional consequence (**e**) (SIFT score less than 0.05 (ref. ^24^)) and proportion of the genome in extended homozygosity (**f**) (ROH more than 1 Mb). Outside Oceania, values are for the population indicated, with continental distributions summarized by black box plots (median (line), upper/lower quartiles (box) and 1.5× interquartile range (whiskers)). Values before masking (dashes) and rescaled after masking (circles) are shown for individuals with more than 5% ancestry other than Indigenous ancestry. ROH estimated from unmasked data and therefore not rescaled. ROH values for individuals with more 5% ancestry other than Indigenous ancestry shown as dashes. (Tiwi *n* = 48, Galiwin’ku *n* = 38, Titjikala *n* = 13, Yarrabah *n* = 45, PNG (HL) *n* = 25, PNG (Is.) *n* = 35, YRI *n* = 108, STU *n* = 102, GBR *n* = 91, CHS *n* = 105, PEL *n* = 85). **g**, Variant discovery with increasing sample size per population, averaged (ten replicates). Yarrabah and PNG (Is.) excluded because of missing data after ancestry masking. **h**, Novel variant discovery per continent after sampling 80 individuals from each of the other continents, averaged (ten replicates). 1000 Genomes codes: YRI, Yoruba, Africa; PEL, Peruvian, America; STU, Sri Lankan, South Asia; GBR, British, Europe; CHS, Southern Han Chinese, East Asia.

To compare the proportions of novel and geographically restricted variation across populations, we analysed equal subsamples of five unrelated individuals from each of the 32 populations in our cohort and the 1000 Genomes (Fig. 1a,b). This ensured that the smallest sample, Titjikala, was included. As all individuals from Yarrabah have some non-Indigenous ancestry, the five with the least missing data after ancestry masking were selected (for analyses without subsampling or ancestry masking, see Supplementary Fig. 1). The observations below hold for larger subsamples of 15 and 25 individuals per population (Supplementary Fig. 1).

Consistent with previous studies^21,22^, total autosomal variation declines with distance from sub-Saharan Africa. Indigenous Australians and Papuans have the least total variation of any population analysed here, with the largest deficit for variation shared across some but not all continents (Fig. 1a,b), consistent with previous reports showing that the separation of Australians and Papuans predates that of all other populations outside Africa^1^. Indigenous Australians have the highest count of variation that is either private to population or private to continent outside Africa (Fig. 1a,b and Supplementary Fig. 1). This ranges from 7.3% to 9% of SNVs in Oceania, with the next highest (6.1%) the JPT (Japanese in Tokyo, Japan), in East Asia. Interestingly, variation occurs less often as singletons in Oceania, particularly among Tiwi people, with the minor allele frequency spectrum showing more variation at a higher frequency within a population sample than seen in populations of other continents (Fig. 1c).

Indigenous Australians and Papuans have the lowest heterozygosity worldwide (Fig. 1d). Within the region, on average the Tiwi had the lowest genetic diversity and Yarrabah the highest (both before and after the ancestry masking (Fig. 1d)), reflecting the diverse origins of the latter community.

The high levels of population- and continent-private variation in Oceania extend to polymorphisms of potential functional significance. Our cohort lacks phenotypes, so associating genetic variation with diseases relevant to Indigenous communities is impractical, although some observations may be made. Considering coding variation in 32 genes associated with type 2 diabetes^23^, we find 51 non-synonymous variants in Galiwin’ku (other groups are similar). Of these, five are either population-private or private to Oceania. These values are typical for equal sample sizes from Europe, Asia and the Americas (Supplementary Note 3). Genome-wide, people in Oceania also have typical numbers of variants of predicted functional consequence on the basis of sequence constraint (SIFT^24^ and PolyPhen^25^, Supplementary Table 1). However, genomes of people from Oceania have fewer variants annotated as pathogenic or likely pathogenic in the clinical database ClinVar^26^ (Supplementary Fig. 1f and Supplementary Table 1), no doubt because of ascertainment bias in ClinVar. Averaged across each Oceanic population sample, we observe 104 variants (median, range 97–108) designated pathogenic or likely pathogenic in ClinVar (0.00225% of variants), whereas the European samples average 184 (median, range 174–202, 0.00313% of variants).

Of relevance to clinical interpretation of predicted functional variation, Indigenous Australians and Papuans have the highest proportions on average of their genomes in runs of homozygosity (ROH; Fig. 1f and Extended Data Fig. 1b,c). Individual values are typically more extreme than those of the Indigenous American peoples (PEL) from Peru, a largely unadmixed population with a low long-term effective population size^14^ and reduced heterozygosity consistent with serial founder events^2^. For example, Tiwi genomes typically exceed 10% extended homozygosity, three times that of Indigenous American peoples (Fig. 1f) and ten times that of Eurasian populations. This extended homozygosity is consistent with elevated background relatedness, probably because of a low long-term effective population size, rather than consanguinity, which is often observed in population isolates^27^ (Extended Data Fig. 1b). Variation with predicted functional consequence more likely occurs in the homozygous state in Oceania than elsewhere (Fig. 1e).

## Sample size and variant discovery

The distribution of variation will affect studies of disease genetics in Indigenous populations. Although the engagement of communities with genomic studies is their choice^28^, our results inform the design of sampling approaches to maximize recovered diversity. To understand the sample size required to adequately capture common variation in Indigenous Australian populations, we calculated variant discovery with progressively increasing sample size^29^. Despite having the highest levels of population-private variation outside Africa (Fig. 1a), the discovery of this variation saturates at much lower sample sizes than for populations on other continents (Fig. 1g). Although the 1000 Genomes populations continue to reveal more variants with increasing sample size, partly because of the steady accumulation of rare variants (including singletons), the number of new variants added by each additional genome of individuals from Oceania diminishes more rapidly. This is consistent with the skewed allele frequency spectra in these samples (Fig. 1c) and indicates relatively small effective population sizes.

Even at small sample sizes, individuals from Oceania have substantial uncharacterized variation. After sampling 80 individuals from each of the other continents, we tested how much novel variation was recovered when sampling within each continent (Fig. 1h). This revealed rates of novel variant discovery in Oceania similar to those in East and South Asia, up to a sample of around 30, much greater than the rates of either Europe or the Americas (this is probably affected by admixture of people from Europe with those from the Americas).

## Population structure

Although the sample sizes required for an Indigenous Australian genomic reference panel are probably small, the breadth of communities to include will depend on population structure across the continent. Population structure arises when non-random mating produces systematic differences in allele frequencies between subsets of a larger population. The nature and strength of such structure is typically a consequence of demographic processes such as isolation, population divergence times, historic effective population sizes and migration rates. Understanding structure is fundamental for studies of demography and disease^30,31^.

**Fig. 2:**
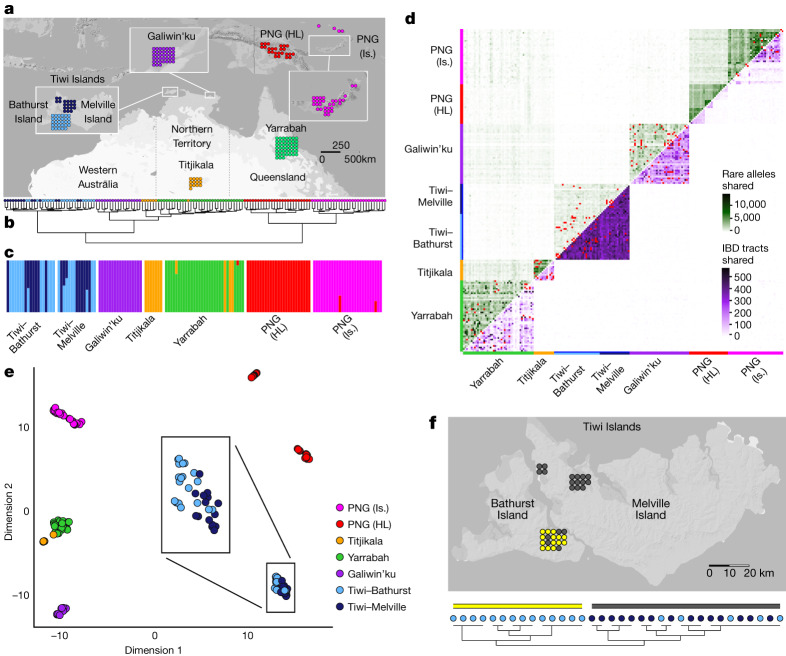
Population structure. **a**, Location and sample size for all Australian and Papuan samples. **b**, Hierarchical clustering of unrelated individuals on the basis of pairwise outgroup *F*_3_ statistic values. Colour corresponds to sampling location. **c**, ADMIXTURE-inferred ancestry for unrelated individuals allowing seven clusters, ordered according to sampling location. Colour was assigned to each cluster post hoc on the basis of the scheme in **a** and the majority membership of each cluster. **d**, Pairwise sharing of rare alleles (above diagonal) and IBD (below diagonal) tracts among all individuals. Counts were rescaled according to the proportion of the genome missing due to ancestry masking in each pairwise comparison. Comparisons between first- and second-degree relatives are indicated in red. **e**, UMAP clustering of unrelated individuals on the basis of minor allele frequency-corrected COV distances, reduced to the first ten components by MDS. Box expands the positions of Tiwi Island individuals. **f**, Clustering of Tiwi individuals on the basis of co-ancestry values estimated using fineSTRUCTURE run on all unrelated and unadmixed samples (see Extended Data Fig. 4a for the full tree). Light blue (Bathurst Island) and dark blue (Melville Island) indicate sampling location, and yellow and grey indicate cluster membership.

**Extended Data Fig. 2:**
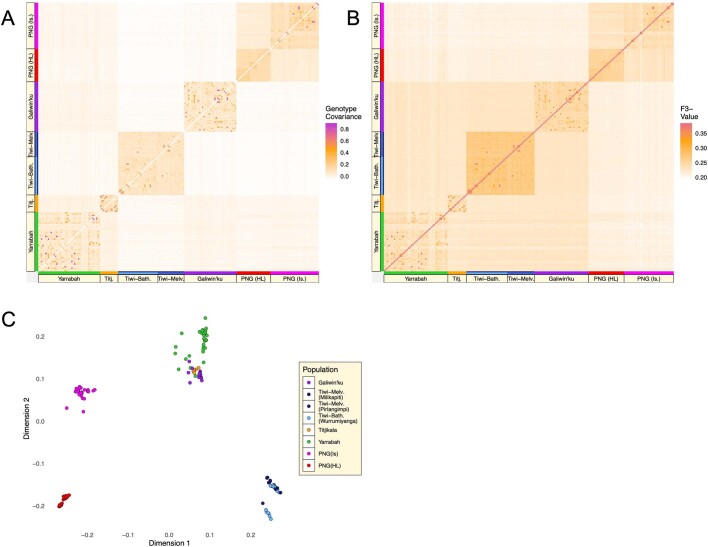
Genetic distances and population structure within Oceania. **A**. Heatmap of the minor allele frequency corrected pairwise covariance (COV) values between all individuals in the NCIG + PNG (masked) dataset (including related individuals). Individuals are listed in the same order along each axis and the population they were sampled from is indicated along the axes. Note that higher values of genotype covariance indicate greater genetic similarity. **B**. Heatmap of pairwise outgroup F_3_-statistics of the form F_3_(YRI;AUAx,AUAy), where AUAx and AUAy are any pair of individuals from the NCIG + PNG (masked) dataset (including related individuals). Individuals are listed in the same order along each axis and the population they were sampled from is indicated along the axes. Higher values indicate greater genetic similarity. **C**. Multidimensional scaling applied to the pairwise genotype covariance (COV) matrix estimated from the *NCIG* + *PNG (masked)* dataset after filtering to unrelated individuals. The first two dimensions are shown. (see Methods for further details of all three plots).

**Extended Data Fig. 3:**
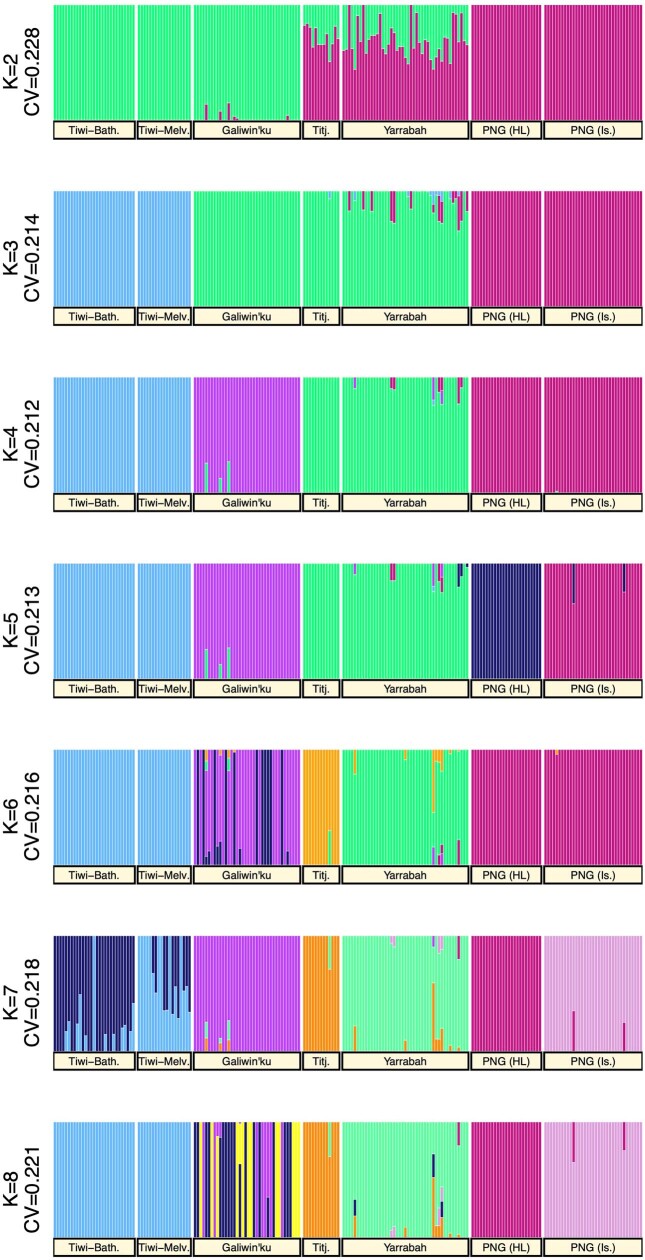
ADMIXTURE ancestry assignment. The clustering algorithm ADMIXTURE applied to the NCIG + PNG (masked) dataset (including related individuals) assuming K = 2 to K = 8 clusters (subpopulations) are represented in the data (See Methods). Clustering makes no reference to the sampling locations of the individuals and is based on genetic data alone. Individuals are listed along the x-axis, grouped according to their sampling location, with bars above reflecting their cluster assignment in the following manner: each inferred cluster is labelled by a colour and the proportion of bar assigned that colour represents the probability that the individual is assigned to that cluster. Colours were manually selected (*post-hoc*) for K = 7 to match the labels in panels 2 A and 2B of Fig. 2, and for other values of K the colouring scheme was merged or split as appropriate. Also shown are the cross-validation (CV) scores used for model selection.

**Extended Data Fig. 4:**
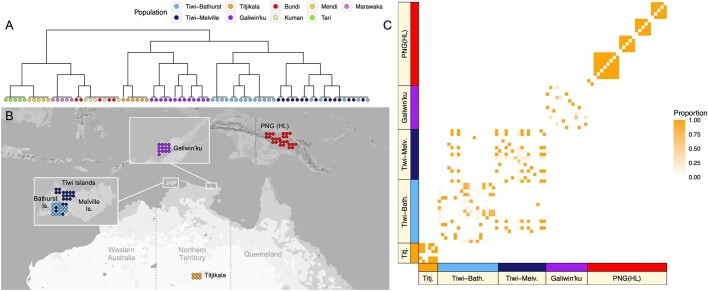
Fine-scale genetic structure within Oceania. **A**. Hierarchical clustering tree (up to 13 clusters) produced from the maximum *a posteriori* state partitions inferred by fineSTRUCTURE (see Methods). The NCIG + PNG (masked) dataset used for this analysis was reduced to a subset of unadmixed and unrelated samples. Samples are coloured according to their sampling location. Note that the 25 samples from Papua New Guinea are denoted by their sub-sampling locations in this analysis (Bundi, Kundiawa (Kuman), Marawaka, Mendi and Tari). **B**. Clustering of the NCIG + PNG (masked) dataset into 5 clusters based only on genetic data using fineSTRUCTURE. The dataset used for this analysis was reduced to a subset of unadmixed and unrelated samples. For each individual, the coloured symbol represents the genetic cluster to which the individual is assigned. **C**. The fineSTRUCTURE coincidence matrix showing the proportion of cluster partitions in which two individuals are grouped in the same cluster during the MCMC. The NCIG + PNG (masked) dataset used for this analysis was reduced to a subset of unadmixed and unrelated samples.

Applying a range of methods, we detect structure and classify individuals into clusters that coincide extensively with their geographical origin (Fig. 2, Extended Data Figs. 2–4 and Supplementary Note 4). More precisely, hierarchical clustering of pairwise outgroup *F*_3_ statistics (Fig. 2b), ADMIXTURE^32^ (Fig. 2c) and fineSTRUCTURE^33^ (Fig. 2f) cluster individuals. Elsewhere, the geographical labels coincide strongly with the discriminating measures of the analysis. In each analysis, the overwhelming majority of individuals are assigned to ‘correct’ (geographically defined) groups, and for the Tiwi (uniform manifold approximation and projection (UMAP)^34^ and fineSTRUCTURE) and PNG Highlands (HL) (UMAP), groups are assigned at fine geographical scales of as little as tens of kilometres. Except for four individuals from Titjikala and Yarrabah, hierarchical clustering and ADMIXTURE-inferred groups coincide with geographical labels, and fineSTRUCTURE is concordant for all individuals analysed (Fig. 2f and Extended Data Fig. 4). These methods infer a bifurcation between Australian and Papuan groups, followed by the divergence of the Tiwi—the only Australian group to speak a non-Pama–Nyungan language (Fig. 2b,c and Extended Data Fig. 3). Rare allele and identity-by-descent (IBD) tract sharing between individuals from the same region is higher than for individuals from different groups, revealing strong within-sample homogeneity (Fig. 2d).

**Extended Data Fig. 5:**
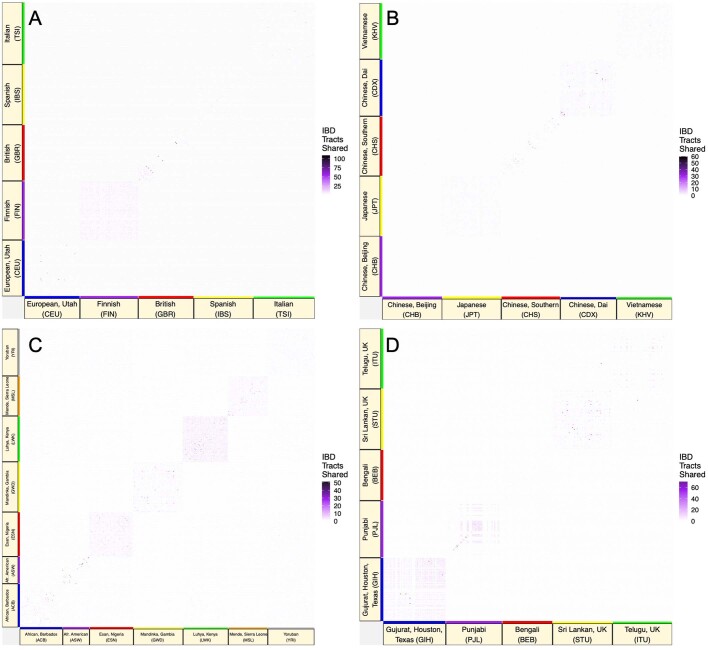
**Global IBD tract sharing**. Heatmaps depicting the number of tracts shared IBD (inferred using the RefinedIBD algorithm; see Methods and Supplementary Note 4) within four continental samples from the 1000 Genomes collection: **A**. Europe, **B**. East Asia, **C**. Africa and **D**. South Asia. A comparable plot featuring NCIG samples is presented in Fig. 2d of the Main Text. Comparisons of samples inferred to share a familial relationship (2nd degree or closer) were masked in red. Note that a different scale was used for each heatmap to maximise definition.

**Extended Data Fig. 6:**
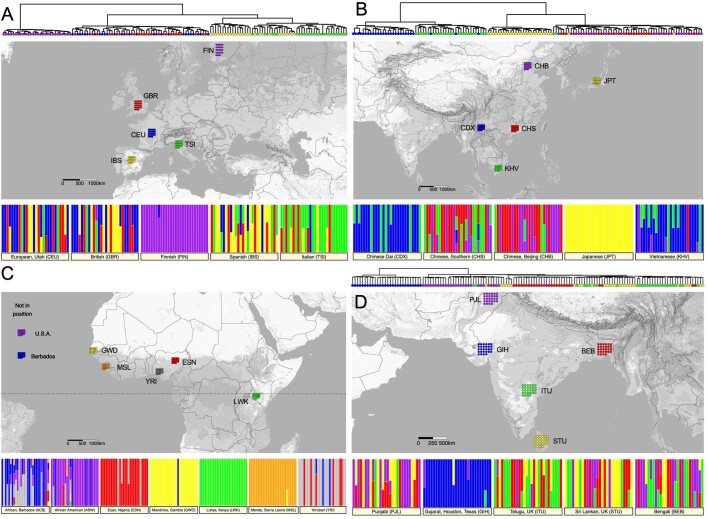
**Global population structure**. Results of the ADMIXTURE algorithm and hierarchical clustering of outgroup F_3_-statistic values for four continental samples from the 1000 Genomes collection; **A**. Europe, **B**. East Asia, **C**. Africa and **D**. South Asia. The maps depict the sampling locations for each population, in addition to the sample size used (*n* = 28 per population). Note that approximate locations for some populations (i.e. CEU, ITU and STU) are given as per the original 1000 Genomes publication^14^. Coloured tip-points below each leaf of the hierarchical clustering tree depict the geographic population label of the individual (from the maps). Hierarchical clustering was not performed on African samples due to the use of Yoruba as the outgroup population (see Methods). The bar charts show the output of the clustering algorithm ADMIXTURE applied to each sample, assuming the same number of clusters as the geographically defined samples (K = 5 for Europe, East Asia and South Asia, and K = 7 for Africa). Clustering makes no reference to the sampling locations of the individuals and is based on genetic data alone. Individuals are listed along the x-axis, grouped according to their sampling location, with bars above reflecting their cluster assignment in the following manner: each inferred cluster is labelled by a colour and the proportion of bar assigned that colour represents the probability that the individual is assigned to that cluster. Colours were manually selected (post-hoc) to match the labels in the maps. See Fig. 2 of the main text for the results of these same algorithms when applied to the NCIG + PNG dataset.

The complex population structure shows that Indigenous Australians form neither a single genetic population nor one with PNG. Rather, we observe a striking, previously undescribed pattern of regional differentiation. For context, we apply several of the same methods to the 1000 Genomes continental groups (Extended Data Figs. 5 and 6). Although these have undergone different demographic events, including expansions and large-scale admixture^19,35^, subsamples have similar geographical separation. ADMIXTURE and hierarchical clustering do not group Europeans, East Asians and South Asians with the same accuracy as populations in Oceania (Extended Data Fig. 6), which have a structure more like that of sub-Saharan Africans. Pairs of individuals in Eurasian populations typically share fewer than five IBD tracts longer than 1.5 cM (Extended Data Fig. 5), an order of magnitude smaller than typical in Australian populations (Fig. 2d).

**Extended Data Fig. 7:**
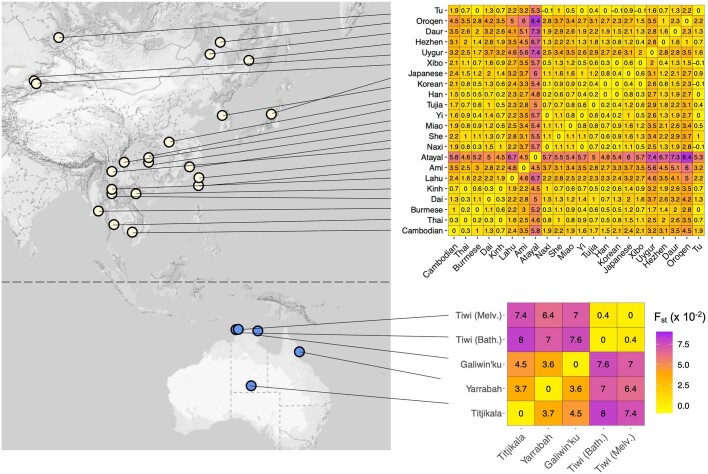
Pairwise F_ST_ between Australian, PNG and Asian populations from the SGDP. Genetic differences between Indigenous Australian communities are significantly greater than between groups from other continents distributed over a comparable geographic range. Heatmaps show pairwise F_ST_ differences between all East Asian populations, and all Australian communities. Note, for instance, F_ST_ between Galiwin’ku and Titjikala (0.045), is as high as between Cambodia and Oroqen (0.045), groups separated by three to four times the geographical distance. All F_ST_ values were calculated on a set of variants polymorphic in an African outgroup population (Mbuti), thus providing an unbiased estimator of F_ST_. Colour scales for both heatmaps are the same.

Pairwise fixation index (*F*_ST_) estimates for populations in Australia compared with those between Simons Genome Diversity Panel (SGDP) populations (Extended Data Fig. 7) further support a scale of population structure in Australia that is among the strongest seen between human populations sampled from the same continent. Taken together, our results demonstrate that it is vital to broadly sample Indigenous Australian and Papuan populations for clinical applications and for characterizing the full spectrum of human genetic variation.

## Relationship to PNG

The strength of structure within Australia and to PNG shows that samples from PNG (which contribute to gnomAD via the HGDP collection) are an inadequate reference for variation in Australia. To understand whether the relationship to PNG is uniform across all Australian populations, we use *F* statistics^36^, measures of shared genetic drift, to explore potentially subtle differences in allele sharing with PNG.

**Fig. 3:**
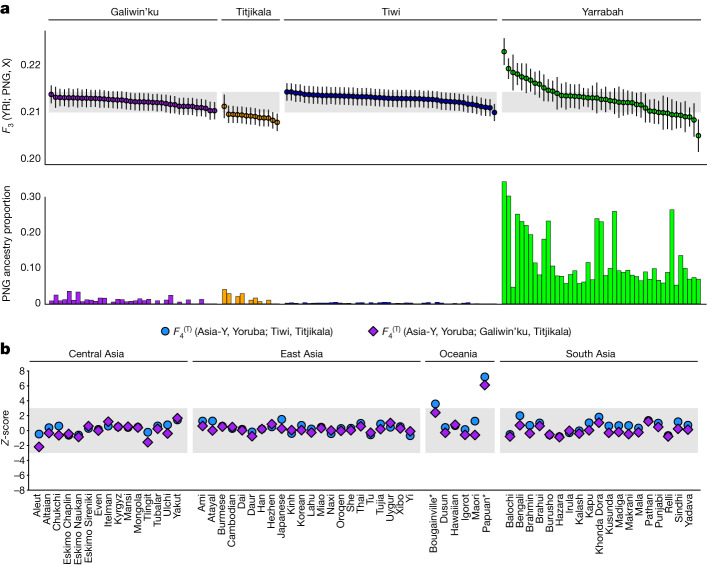
Historical relationships between Australian and PNG populations. **a**, Top, shared genetic drift between populations estimated by outgroup *F*_3_ statistics of the form *F*_3_ (Yoruba; PNG, *X*), where *X* is an Australian individual. Higher values indicate greater shared genetic drift with PNG. Individuals are rank-ordered by *F*_3_ value within populations, with block jackknife-estimated standard errors shown as vertical bars. The range of *F*_3_ values for individuals in the Tiwi and Galiwin’ku population samples is indicated by horizontal shading. Bottom, the proportion of Papuan global ancestry (after masking) estimated by RFMIX for the same individuals. These per-individual metrics include related individuals. Sample sizes: Tiwi *n* = 48, Galiwin’ku *n* = 38, Titjikala *n* = 13, Yarrabah *n* = 45. **b**, *Z-*scores derived from *F*_4_ statistics of the form ${F}_{4}^{({\rm{T}})}$(Asia-*Y*, Yoruba; Australia-*X*, Titjikala), where Asia-*Y* is a Eurasian or Oceanic population sample from SGDP and Australia-*X* is either the Galiwin’ku or Tiwi Islands sample. *Z-*score values greater than 3 provide statistically significant evidence that population Asia-*Y* shares more genetic drift with Tiwi/Galiwin’ku than with Titjikala, and these populations are marked with an asterisk. The per-individual metrics include related individuals.

**Extended Data Fig. 8:**
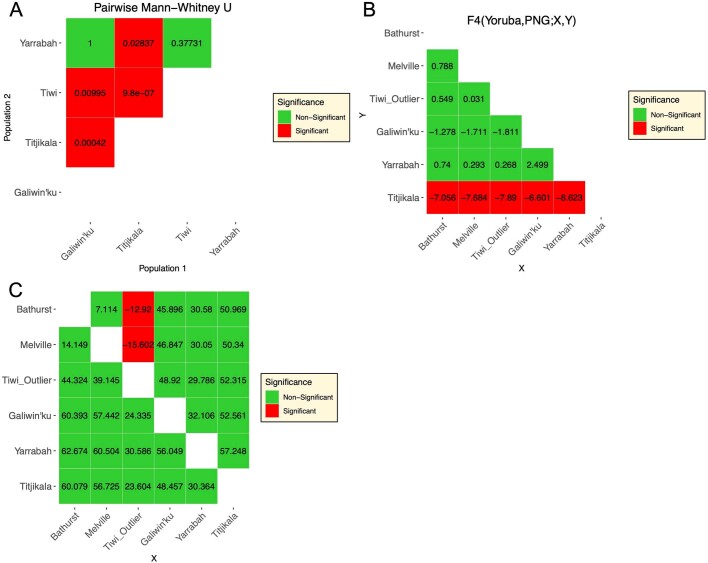
Comparing genetic drift shared with PNG using F-statistics. **A**. p-values from a Mann Whitney U test performed pairwise between the Australian samples grouped by community. Here we assume that the outgroup F_3_ statistic for each individual (relative to PNG) in Fig. 3a is drawn from a common distribution for each community. The distributions of the statistic for a pair of communities are compared using the Mann Whitney test. Significant p-values (less than 0.05; shown in red) indicate the null hypothesis, that the distributions of the statistic for each group are equal, has been rejected. A two-sided test was used, with the Bonferroni p-value adjustment method. **B**. Matrix of all pairwise ${{\rm{F}}}_{4}^{({\rm{T}})}$ statistics (calculated using ADMIXTOOLS) of the form ${{\rm{F}}}_{4}^{({\rm{T}})}$ (YRI, PNG; X, Y), where ‘X’ and ‘Y’ are any one of the Australian populations in the NCIG dataset. Here we separate the Tiwi samples into the islands they are sampled from. Numbers reported are Z scores (the default ADMIXTOOLS output) and are significant when they exceed +3 or −3. See Methods for description of ‘Tiwi_Outlier’ label. **C**. Table showing all possible F_3_ statistics of the form F_3_(AUAx; PNG, AUAy), where ‘AUAx’ and ‘AUAy’ (simply labelled ‘X’ and ‘Y’ in this figure) are a pair of groups from the NCIG dataset. Here we separate the Tiwi samples into the two islands, Bathurst and Melville, and we also treat the Tiwi Outlier individuals as a separate group (See Methods for a description of the Tiwi Outlier individuals and a justification for removing them from our main analyses due to evidence of substantial recent admixture with PNG in their genomes). Text within each cell is the Z*-*score for the F_3_ statistic from a block jackknife (directly from the software Admixtools). Following the theory of Patterson et al. (2012), statistically significant evidence of admixture between PNG and AUAx, but not AUAy, is indicated by a Z-score lower than −3, here indicated by red.

We find significant differences (Kruskal–Wallis omnibus test) between Australian populations in their shared drift with PNG (Fig. 3a; outgroup *F*_3_ statistics). Samples from Titjikala share less drift with PNG than those from Tiwi or Galiwin’ku and most samples from Yarrabah, and the Titjikala samples are not derived from the same distribution as the other samples (pairwise Mann–Whitney *U-*tests; Extended Data Fig. 8a). Yarrabah individuals have highly variable *F*_3_ statistics, correlated with the degree of recent PNG-related ancestry inferred in each genome (Fig. 3a; Spearman’s correlation coefficient permutation test *P* = 0.017). Although several scenarios, explored below, could result in these patterns, this excludes a single division of ancestral Australian and Papuan populations without subsequent genetic interactions.

We calculate ${F}_{4}^{({\rm{T}})}$ statistics of the form ${F}_{4}^{({\rm{T}})}$ (YRI, PNG; Australia-*X*, Australia-*Y*) to formally test for a non-cladistic relationship between Australian and PNG populations. We reject the null hypothesis that Australian populations form a clade (that is, are equally related) with respect to PNG for every combination pairing Titjikala with another Australian group (Extended Data Fig. 8b), confirming the three northern populations share more genetic drift with PNG, contrary to previous reports^1,6^. Although this and previous studies infer recent PNG or Torres Strait Island-related ancestry in North Queensland (here Yarrabah)^1,17,37^, we find no evidence of recent admixture from a PNG-related source population into Tiwi or Galiwin’ku (Extended Data Fig. 8c). By expanding the *F*_4_ analysis to include more Asian and Oceanic populations (SGDP^6^), we rule out common admixture from an external source population (for example, from Island Southeast Asia) into both PNG and Tiwi and/or Galiwin’ku, explaining the elevated shared drift with PNG (Fig. 3b). The remaining plausible demographic scenario is an extended period of genetic interaction between the ancestral populations of PNG and northern Australia once structure began to form within Australia. Differential ancestry from extinct hominin groups may also have affected these patterns but was not investigated.

**Extended Data Fig. 9:**
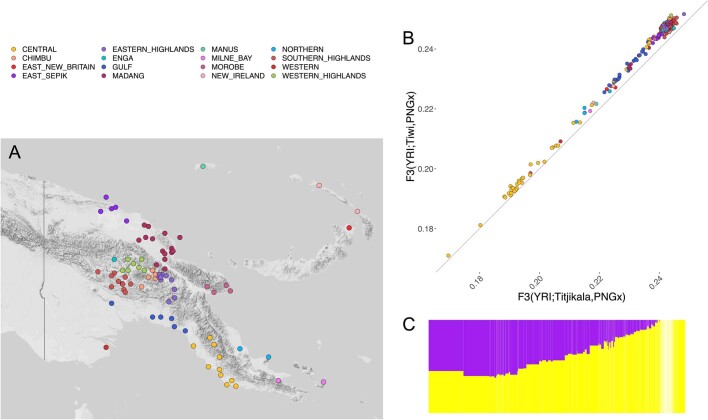
Shared genetic drift between Indigenous Australian communities and a panel of Papuan populations. (Left) Map showing the locations of all populations sampled in the dataset of Bergstrom et al. (2017), with colour code indicating the regional province. (Top Right) Scatterplot of values of outgroup F_3_ statistics of the form F_3_(Yoruba; Titjikala, PNG-X) versus F_3_(Yoruba; Tiwi, PNG-X), where ‘PNG-X’ is a PNG individual in the dataset described by Bergstrom et al. (2017). Colours represent the sampling location of the PNG individual (see map to the left). (Bottom Right) ADMIXTURE barplot showing putative PNG (yellow) and non-PNG (purple) global ancestry estimates for each of the individuals in the above scatterplot. Individuals in the barplot are shown in the same order left to right as in the scatterplot.

To assess whether shared drift with Australian populations is uniform across PNG, we calculate outgroup *F*_3_ statistics using genotype data for a larger collection of individuals from PNG (Extended Data Fig. 9). The values are uniformly higher for Tiwi than Titjikala across all regions, showing that genetic interaction between northern Australia and PNG ceased before structure developed within PNG or that any early structure within PNG was erased by later migrations. We note that this analysis only considers groups from the east of the island of New Guinea.

## Historical relationships in Australia

**Fig. 4:**
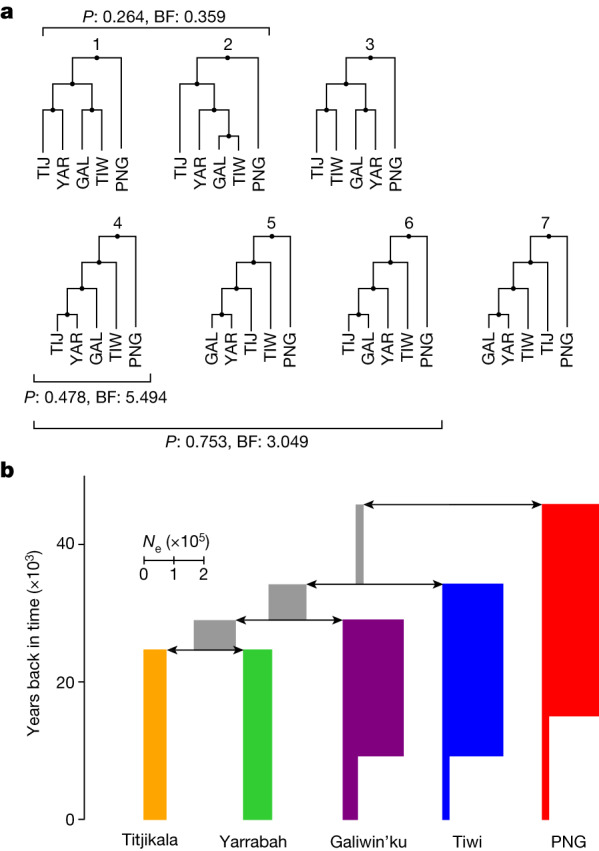
Historical relationships and processes that have shaped genomic variation in the sample. **a**, Seven plausible population histories were included in ABC simulations, in which effective population sizes and migration rates were allowed to vary. Also shown is the evidence for each scenario or group of scenarios. **b**, Parameter estimates for split times and effective population sizes for the most likely single scenario, scenario 4 (see Supplementary Figs. 2–4 for parameter distributions). BF, Bayes factor; GAL, Galiwin’ku; *N*_e_, effective population size; *P*, posterior probability; PNG, Papua New Guinea (HL); TIJ, Titjikala; TIW, Tiwi Islands; YAR, Yarrabah.

The relative importance of the heterogeneous relationships to PNG depends largely on demographic parameters, including effective population sizes, split times and migration rates within Australia. We apply an approach combining efficient simulation of genetic data^38^ with approximate Bayesian computation (ABC)^39^ to evaluate evidence for each of seven plausible phylogenetic topologies. Modelling several migration parameters, we assess the contribution from PNG to each Australian population and between Australian populations over time (Fig. 4a,  Methods and Supplementary Note 5). The topology with the most support (scenario 4, Fig. 4a) and the most-supported combination (scenarios 4–6, Fig. 4a) have the Tiwi as an outgroup to the other Australian groups, supporting a division on the basis of language family rather than geographical distance. This was confirmed by an alternative approach, AdmixtureBayes^40^ (Supplementary Fig. 5; 37.6% of sampled trees have the Tiwi as an outgroup).

Both methods support Galiwin’ku as an outgroup to Titjikala and Yarrabah (32.7% of AdmixtureBayes-sampled trees). However, our ABC analysis cannot rule out some alternatives (scenarios 5 and 6), and AdmixtureBayes supports a star-like consensus topology with extremely short internal branch lengths and long terminal branches (Supplementary Fig. 5). We formally tested whether Tiwi and Galiwin’ku, the two geographically closest communities, form a clade with respect to the other Australian groups (scenarios 1 and 2) but found little evidence to support this (Fig. 4a).

On the basis of the best-supported topology (Fig. 4b), we used our ABC method to estimate split times (Supplementary Table 2 and Supplementary Fig. 2), effective populations sizes (Supplementary Table 3 and Supplementary Fig. 3) and migration rates (Supplementary Fig. 4). We infer that the split between Indigenous Australians and Papuans occurred 1,636 generations ago (47 ka, highest 95% posterior density interval 27–64 ka). This is older than the previous estimate of 37 ka from autosomal data^1^ but consistent with estimates from mitochondrial DNA^7^. This ancestral Australian population existed for 12,000 years with a small but statistically well-supported effective population size of around 2,000 (median; Supplementary Fig. 3 and Supplementary Table 3), followed by the relatively rapid separation of the ancestral populations of Tiwi (35 ka) and Galiwin’ku (31 ka) and a Titjikala–Yarrabah split at 26 ka.

The early and rapid division of Australian groups inferred via ABC, the star-like consensus topology inferred by AdmixtureBayes and the Multiple Sequentially Markovian Coalescent-2 (MSMC2) analysis presented below imply that the history of these populations probably involved a complex period of overlapping and incomplete isolation. However, once isolation was established, the methods infer limited migration between groups, although we caution that there is poor inference of historic migration rates with ABC (Supplementary Fig. 4), and no admixture events were inferred in the 15 top-ranked AdmixtureBayes trees (Supplementary Note 5).

Notwithstanding these findings, several lines of evidence (Supplementary Note 5) are consistent with recent Papuan or Melanesian ancestry in individuals from Yarrabah. Explicitly modelling this in the last three to seven generations gave strong support for a 1.8% contribution from PNG or a PNG-proximal population into the current Yarrabah population (Supplementary Table 2 and Supplementary Fig. 4). With this exception, combining the evidence presented here with the strong population structure observed above indicates that long-term migration between populations was limited relative to other global populations. We note that these inferences, on the basis of genetics, can also be strongly informed by community knowledge and history.

## Effective population size

Using the ABC model, we infer that the Tiwi, Galiwin’ku and PNG populations underwent historic changes in effective population sizes, with strong support for an extended period of large effective population sizes, 10,000 for Galiwin’ku and 7,000 for Tiwi, before undergoing a strong reduction (Supplementary Fig. 3). The approach gives poor resolution on the time of these events, so we apply two methods that leverage historic recombination events to infer effective population size: over the last few hundred generations (IBDNe)^41^ and deeper in time (MSMC2)^42^.

**Fig. 5:**
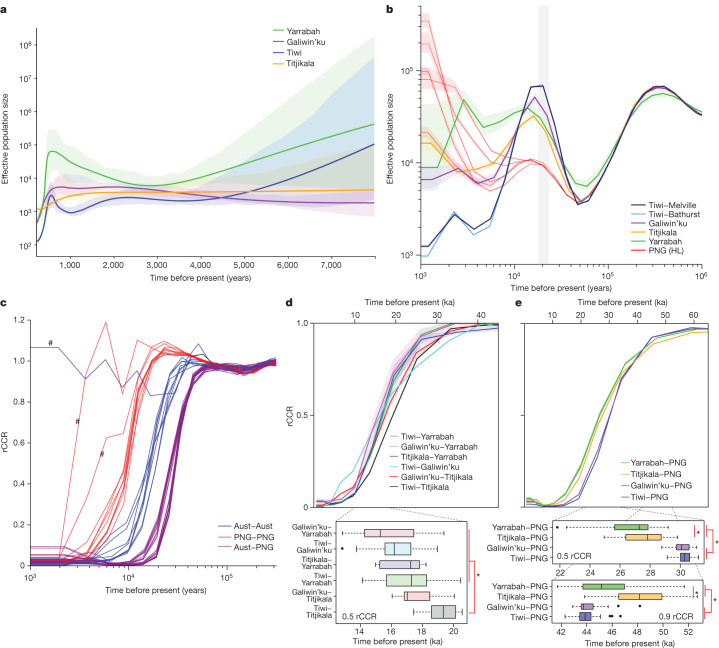
Effective population sizes and population isolation. **a**, Mean effective population size estimates using the IBDNe algorithm. Shading indicates 95% bootstrap confidence intervals. **b**, Effective population size estimates for Australian and PNG (HL) populations inferred using MSMC2 from eight phased haplotypes (four individuals) per population. The line and shading are the mean and s.e.m. of five replicates randomly selected from each population sample. Grey bar indicates the Last Glacial Maximum (21 ± 3 ka). **c**, rCCRs for all 45 possible population pairs (5 Australian + 5 PNG (HL)) estimated with MSMC2. Each line represents the mean rCCR of ten selected sets of eight phased haplotypes (2 haplotypes × 2 individuals × 2 populations). An rCCR of 1 indicates a single ancestral population. An rCCR of 0.5 is a common heuristic indicating the point of population separation. The relative shape of rCCR curves reflects different separation dynamics such as post-split gene flow^44^. Hash indicates three geographically close population pairs (Mendi–Tari, Bundi–Kundiawa in PNG and Bathurst–Melville in Tiwi) that show recent or incomplete separation. **d**, rCCRs for population pairs within Australia (with Tiwi samples combined), showing mean (line) and s.e.m. (shading) for 10 replicates. Lower box plots show the estimated times of population separation (rCCR = 0.5). Asterisks indicates a significant difference between the Tiwi–Titjikala and all but one of the other separation times. **e**, rCCRs for population pairs between Australia and PNG (with PNG samples combined) showing mean (line) and s.e.m. (shading) for 10 replicates. Lower box plots show the estimated times of population separation (rCCR = 0.5) and of the onset of population structure (rCCR = 0.9). Asterisks indicate significant differences. All box plots display the median rCCR across 10 replicates (line), upper and lower quartiles (box), 1.5× interquartile range (whiskers) and outliers (points).

The past 6,000 years are characterized by small but stable effective population sizes ranging from around 10,000 for Yarrabah (likely inflated by the diverse origins of this community) down to 1,500 for the Tiwi Islands, a value consistent with historical surveys of the census population size^12^ (IBDNe; Fig. 5a, Supplementary Fig. 6 and Supplementary Note 6). We infer a marked decline in population sizes over the past few hundred years, although this is less evident for Titjikala. This contrasts with Eurasian populations, which have had steady population growth over the past 8,000 years, with a rapid increase in the past 1,000 (refs. ^19,41,43^).

The relatively small recent effective population sizes estimated across Australia were preceded by dramatically larger values 15–20 ka (MSMC2; Fig. 5b and Supplementary Note 6). After a common bottleneck 50–60 ka, as seen in all populations outside Africa^1,19^, the Australian and Papuan populations grow until about 20 ka, resulting in markedly larger values for all four Australian populations (particularly Tiwi) than seen in PNG. They then decline, coincident with the end of the Last Glacial Maximum. These values are broadly consistent with those obtained using the ABC modelling above.

## Population isolation

We use MSMC2 (refs. ^42,44^) (Supplementary Note 6) to explore the timing and dynamics of population separation via the relative cross coalescence rate (rCCR). Between-population rCCR curves show three distinct clusters (Fig. 5c) indicating that the ancestral Australian and Papuan populations were genetically isolated by 27–30 ka, at least 10,000 years earlier than the establishment of population structure within Australia, which in turn is 5,000–10,000 years earlier than the separation of the ancestral Highland Papuan populations: values consistent with the ABC analysis. The shape and midpoints of the rCCR curves reveal interesting heterogeneity. In Australia, the oldest separation observed is between Tiwi and Titjikala (19 ka), significantly earlier than the separation of other population pairs (Fig. 5d).

We also observe a complex and heterogeneous pattern of isolation between the ancestral Australian and Highland Papuan populations (Fig. 5e). Considering a rCCR value of 0.9 as a proxy for the initial onset of population structure, Titjikala begins isolation from PNG more than 4,000 years earlier than Tiwi or Galiwin’ku, consistent with the above modelling. This pattern then inverts, with the two northern populations becoming fully isolated from PNG more than 2,000 years earlier than Titjikala.

These non-uniform and non-overlapping isolations within Australian and between Australia and PNG show that the establishment of population structure was complex. A likely scenario, consistent with patterns of shared genetic drift (Fig. 3) and demographic modelling (Fig. 4) is that the ancestral populations of both Tiwi and Galiwin’ku remained in genetic contact with the PNG population for a significant period after they had begun to undergo isolation from the Yarrabah–Titjikala population.

## Mitochondrial diversity

**Extended Data Fig. 10:**
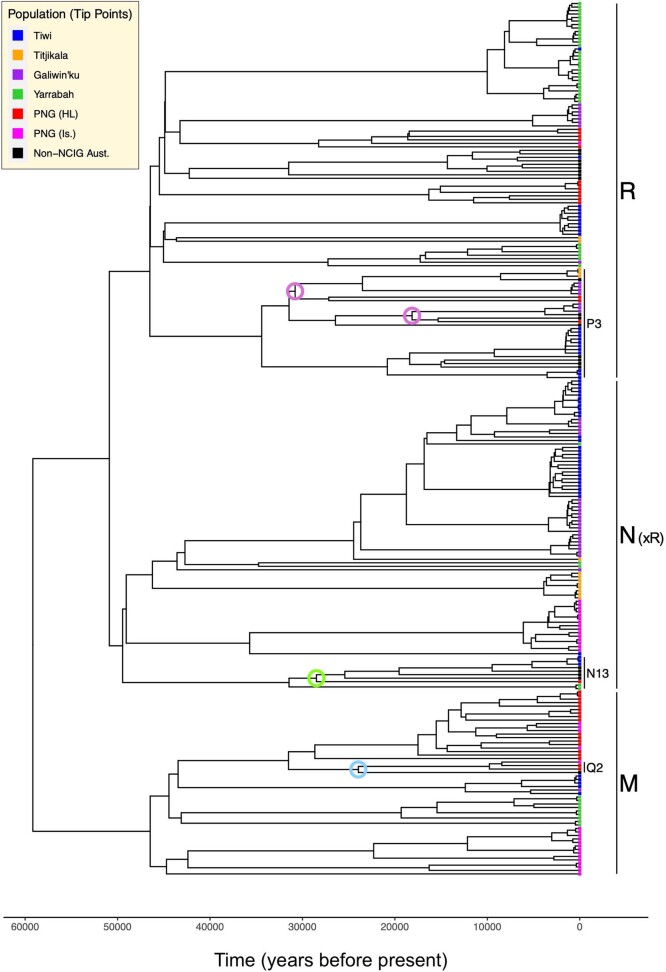
**Mitochondrial phylogenetics**. Population Mitochondrial DNA phylogeny of all individuals from the ***NCIG*** + ***PNG*** dataset, plus additional sequences from GenBank (see Methods and Supplementary Note 7 for samples used and phylogenetic methods). Tip-point labels indicate the community the individual was sampled from. Coloured circles over nodes indicate coalescence events between PNG and Indigenous Australian haplotypes which date to within the last ~35 ka. Clade labels of sub-lineages (P3, N13 and Q2) mark the lineages involved.

**Extended Data Fig. 11:**
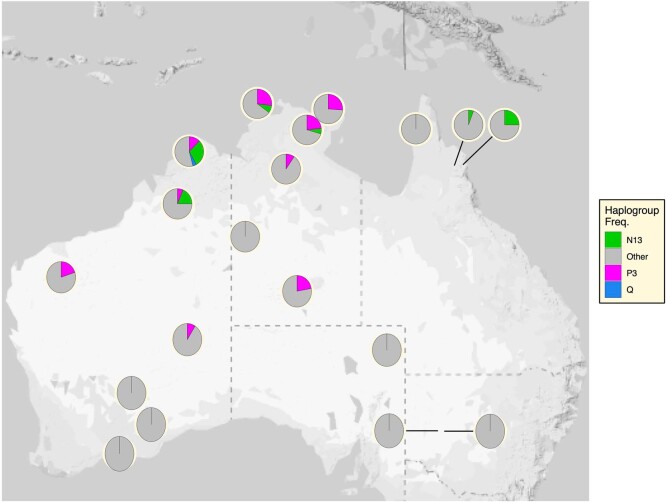
Map with pie charts showing frequencies of the P3, N13 and Q2 Mitochondrial haplogroups. Map with pie charts showing frequencies of the three haplogroups (P3, N13 and Q2), with recent (~35 ka) TMRCA to Melanesian sister lineages in Indigenous Australian communities from both the NCIG dataset, and previously published studies. Note the apparent enrichment of these haplogroups in the Top End and Kimberley regions of Australia. The P3 haplogroup frequency was scored instead of P3b, as some studies did not genotype to this degree of resolution. The P3 lineage coalesces approximately 35 ka and contains both PNG and Indigenous Australian sub-lineages.

Until recently, it was thought that no mitochondrial lineages coalesce between Australians and Papuans more recently than 40–50 ka (refs. ^7,45^), supposedly reflecting the abrupt divergence of ancestral groups after reaching Sahul (the palaeocontinent that includes Australia and New Guinea). The only exceptions were P3b lineages in individuals with Torres Strait Islander ancestry^37,45^ and a single Q2 lineage from the Kimberley^46^. Recently, two studies incorporating a large collection of mitogenomes of individuals from Oceania reported several other shared Australasian lineages that coalesce more recently than 35–40 ka (refs. ^47,48^). Supporting this, we observe two lineages with appreciable frequency in Australia (P3 and N13) and divergence times from PNG more recent than 32 ka (Extended Data Fig. 10 and Supplementary Note 7). Using established haplogroup frequencies, we note that these lineages are more frequent in northern Australia (Extended Data Fig. 11), supporting the inferences of non-uniform allele sharing between Australian and Papuan groups from *F* statistics and the rCCR in the autosomal analyses above.

## Discussion

The establishment of this genomic collection has involved more than a decade of consultation with Indigenous leaders, recurring engagement with communities and participants to build mutual trust and a common dialogue and placing the data under Indigenous governance and custodianship. The result is a sizeable cohort with substantial Indigenous ancestry across north and central Australia from people from two independent language families. Comparable studies outside Australia have highlighted the rich genetic diversity in Africa; the bottleneck experienced by all populations outside Africa; the early establishment of population structure across Eurasia; a complex pattern of isolation, migration and extinct hominin ancestry; and the recent considerable expansion of several, but not all, populations^49^. These broad demographic patterns underpin recent advances in our understanding of the genetic basis of common diseases and have enabled the development of tools to aid the diagnosis of rare diseases. However, these may not necessarily relate to or be effective for Indigenous Australians^50,51^.

We have shown that Indigenous Australians have strong structure relative to other populations outside Africa. By including populations from northern Australia, we have identified a more complex genetic relationship between Indigenous Australians and Papuans than previously inferred^1^. We found that the Tiwi, the only non-Pama–Nyungan language speakers considered here, developed genetic structure from the ancestors of the other Australian communities well before rising sea levels caused the physical separation of the Tiwi islands. Furthermore, non-uniform patterns of shared genetic drift show that this early period was characterized not by discrete separation but rather by an extended period of continuing interaction between the northern populations of Australia and PNG. This was followed by long-term genetic isolation, little detectable migration and strong fluctuation in effective population size, from very large at the end of the Last Glacial Maximum to small and stable over the past few thousand years.

This history has shaped genomic variation in Australia. The early separation of Australians from Eurasians, followed by large effective population sizes of the ancestral Australian populations, have led to the highest levels of previously undescribed private variation observed outside Africa. Notably, 25% of variants are not present in gnomAD, a database approaching saturation for some classes of variation^52^. We observe a depletion of individual heterozygosity and locally common extended haplotypes generating very high levels of ROH and long segments of IBD between individuals. Strong population structure and extended periods of small but stable effective population size almost certainly underpin these observations, rather than recent consanguinity, as observed in more recent population isolates. Failure to account for these signals may confound genomic analyses such as phasing, imputation and association studies, supporting the inclusion of Indigenous Australians in variant databases and resources including genome assemblies.

In addition to population-level applications, our findings are important for individual genomics, including clinical diagnostics. Here, the elevated homozygosity of apparently novel variants specific to Indigenous Australians may falsely lead to them being prioritized as potentially pathogenic. This has implications for any analyses that make judgements about variation in the absence of established phenotypic manifestations, including preconception carrier screening, prenatal diagnostic testing, newborn screening and the prediction of disease predisposition in asymptomatic people. In practice, this points to the need to include individuals from a diverse range of language families and regions.

The value of population-specific reference resources for clinical research and the benefits of personalized medicine have been demonstrated for European populations^53–55^, which are considerably less strongly structured than the communities analysed here. The NCIG collection includes a small fraction of the linguistic, cultural and likely genetic diversity present across Australia. Our results show that no single genomic resource, based on either this collection or current global samples, can adequately capture the genetic diversity present in Indigenous Australians. Importantly, only a relatively small number of individuals from a much wider breadth of communities will be required to overcome this imbalance in the availability of adequate reference data. Ultimately, the engagement, leadership and self-determination of Indigenous people in and through such genomic data will support transformative insights, empowerment, inclusion and equity.

## Methods

### Inclusion and ethics

The DNA samples analysed in this project form part of a collection of biospecimens, including historically collected samples, maintained under Indigenous governance by the NCIG^11^ at the John Curtin School of Medical Research at the Australian National University (ANU). NCIG, a statutory body within ANU, was founded in 2013 and is bound by the National Centre for Indigenous Genomics Statute (2016, updated 2021). This federal government statute requires a majority of Aboriginal and Torres Strait Islander representatives on the NCIG Board, ensuring Indigenous oversight of the centre’s decision-making processes and activities. The board is the custodian of the NCIG collection.

For this project and future work, culturally appropriate community engagement was undertaken^56^. NCIG engaged with traditional owners, community elders and other community representatives to inform the community about the research. This involved contact with the Shire service manager(s), inquiries with community stakeholders, arranging interpreters, promoting the visit in advance and preparing outreach material, including plain-language project summaries and consent forms.

Initial work focused on informing communities about the existence of the historical collection and seeking advice about its continued maintenance and possible future use. During this process, NCIG sought and received with consent (see below) new samples of blood or saliva from current members of the communities we engaged with (some of whom were part of the historical collection). These new samples form the basis of the dataset analysed herein.

Confidentiality agreements, project information and consent forms were communicated to local community organizations, community leaders and participants by means of a community liaison officer, official translation services, local community translators and a video animation. All individuals provided informed personal consent during community visits between circa 2015 and 2018.

The results contained in this paper were returned to communities and all participants using a plain-language summary of the final draft of this manuscript and workshops (two pending) in communities. The community liaison officer was, and is, available to take questions from all participants and community members. The draft of this Article was also available to those who wanted it.

This work was carried out under ANU ethics protocol 2015/065 and the University of Melbourne Ethics protocol 1852770. Further details are in Supplementary Note 1.

### Sequencing, read mapping and variant calling

Individuals in the cohort provided a sample of blood or saliva from which DNA was extracted. Genomic DNA quantification, library preparation and sequencing were performed by the Kinghorn Centre for Clinical Genomics (Sydney, Australia). Sequencing was carried out on an Illumina HiSeqX with 150 bp paired end reads to a minimum read depth of 30×. Fastq files were obtained with permission for 60 Papuan samples^1,5^.

Read mapping and variant calling was carried out as detailed in Supplementary Note 2 to generate the NCIG + PNG autosomal dataset. As needed, this dataset was combined with the low-coverage 1000 Genomes dataset^14^ and/or the Simons Genome Diversity Panel (SGDP)^6^, subsets of the International Genome Sample Resource (IGSR) collection; SNP array data from Papuan populations^57^; and the high coverage (HC) 1000 Genomes dataset^18^  (see Supplementary Note 2).

High-molecular-weight DNA was extracted from five blood samples, sequenced with Chromium 10x at the KCCG and processed with the Long Ranger WGS software package to generate single-sample phased variant call format files that were used to assess phasing accuracy.

### Haplotype inference

Phasing was performed with ShapeIT (v.2.12, default parameters)^58^ using both the low-coverage 1000 Genomes reference panel and phase informative reads^59^. Linked-read data were used to estimate switch error rates^60^ and select an optimal phasing strategy (Supplementary Note 2).

### Ancestry inference

Global ancestry proportions were estimated in the NCIG + PNG dataset using ADMIXTURE (v.1.3)^32^ after intersecting with the low-coverage 1000 Genomes dataset and thinning for linkage disequilibrium. *K* was varied from 2 to 12 in cross-validation mode with ancestry proportions inferred at *K* = 6 and verified via principal component analysis^61^, *F*_4_ ratios^36^ and RFMIX^62^ (Supplementary Note 2).

Local ancestry was inferred using RFMIX (v.1.5.4) with a reference panel of individuals from the NCIG + PNG dataset inferred to have mainly Indigenous ancestry (Supplementary Note 2) and European, East Asian, South Asian and African individuals from the low-coverage 1000 Genomes dataset (see Supplementary Note 2 for parameters and composition of the reference panel). Genomic coordinates were identified for each individual that demarcate regions where one or both haplotypes were of neither Indigenous Australian nor Papuan ancestry, generating a ‘mask’ coordinate file in BED format and a VCF file with variant calls in these regions set to missing. The mask was used to keep all regions of the genome for which both haplotypes have Indigenous Australian or Papuan ancestry and remove all other regions. We refer to this dataset as NCIG + PNG (masked). This masking pipeline was validated using *F*_4_ ratios, ADMIXTURE and principal component analysis, run with the ‘lsqproject’ feature of the EIGENSTRAT software package (EIGENSOFT v.7.2.1)^61^. This mask removed more than 95% of the genome for five individuals who were not considered in subsequent analysis.

### Kinship inference

A subset of 150 unrelated individuals (97 Australian and 53 PNG), up to second-degree relatives (that is, no second-degree relatives or closer present), were identified using KING^63^ with the ‘--unrelated’ and ‘--degree 2’ options from the NCIG + PNG dataset (without ancestry masking). Downstream analyses of population structure revealed eight Tiwi samples from this subset of 150 to cluster in a pattern consistent with one or more of their ancestors being of non-Tiwi Indigenous ancestry (designated ‘Tiwi outliers’; an additional two ‘Tiwi outliers’ were removed with the relatedness filter (Supplementary Note 2)). Unless otherwise stated, all main analyses were performed on this ancestry-masked, unrelated and non-outlier subsample, which included 142 samples: 89 from the NCIG collection (34 Tiwi, 31 Yarrabah, 17 Galiwin’ku, 7 Titjikala) and 53 from PNG (25 Highland PNG, 28 Island PNG). For comparison, ref. ^1^ analyses 69 Australian samples with similar constraints.

### Genomic variation

To assess variant sharing, the NCIG + PNG (masked) dataset was merged with the high-coverage 1000 Genomes dataset^18^ (both underwent equivalent data processing, including variant quality score recalibration filtering at 99.8), taking the union of sites using the PLINK ‘--bmerge’ command^64^ and removing sites that became triallelic using the ‘--exclude’ command.

Variants were assigned to one of four non-overlapping categories as defined previously^14^; observed in a single-population sample (‘population private’); observed in more than one population sample within a single continent (‘continent private’); observed in several, but not all, continents (‘shared across some continents’); and observed in all continents (‘shared across all continents’).

To allow an unbiased comparison, each population sample was restricted to five unrelated individuals using the PLINK ‘--keep’ command (Yarrabah and Island Melanesia (PNG (Is.)) were restricted to the five least-admixed unrelated individuals). Given the potential of relatedness to reduce the levels of variation in these subsamples, we confirmed that no pairs of individuals within Galiwin’ku, Tiwi, Titjikala and PNG (HL) had detectable relatedness up to the fourth degree (the maximum threshold identified by the KING algorithm). The difficulty of obtaining a subset of both unrelated and unadmixed samples from Yarrabah and PNG (Is.) necessitated the inclusion of two pairs of third-degree relatives from Yarrabah.

Allele frequency reports stratified by population and continent were generated using the PLINK ‘--freq’ command (Fig. 1a,b). This analysis, with equal sample size of *n* = 5, is shown for all populations of the 1000 Genomes dataset in Supplementary Fig. 1a and was repeated on the full dataset (that is, without subsampling individuals) both with ancestry masking (Supplementary Fig. 1b) and without (Supplementary Fig. 1c) and on versions of the masked dataset filtered to a sample size of *n* = 15 and *n* = 25 unrelated samples per population (Supplementary Fig. 1d,e).

The above analysis was repeated after subsetting to only sites classified as ‘pathogenic’, ‘likely pathogenic’ or ‘drug response’ in ClinVar (release 20230514; Supplementary Fig. 1f) and after subsetting to non-synonymous variants within the type 2 diabetes associated genes listed in Tables 2 and 3 of ref. ^23^ (Supplementary Note 3). Coordinates of these genes were obtained from GENCODE Release 37 (GRCh38.p13), and non-synonymous variants within the NCIG + PNG + 1000 G (high-coverage) dataset were identified using VEP^65^.

Minor alleles were defined using the PLINK ‘--recode’ command in the above dataset (restricted to five individuals per population sample), where the minor allele is defined in reference to the whole dataset. The allele count within a population sample was recorded using the PLINK ‘--freq’ command and binned from count 1 (seen once in a set of 10 haplotypes) to 10 (fixed in the sample) to generated allele frequency plots (Fig. 1c).

Per-individual counts of heterozygous sites were produced from the full dataset after ancestry masking (NCIG + PNG (masked) + high-coverage 1000 Genomes), with values rescaled to account for the proportion of the genome ancestry masked in each sample (open circles in Fig. 1d). For individuals with more than 5% ancestry other than Indigenous ancestry, these values were also generated from the unmasked dataset (NCIG + PNG + high-coverage 1000 Genomes) (dashes in Fig. 1d).

Phenotypic impact was predicted for amino acid substitutions in the full dataset (both unmasked and masked) using the VEP ‘--sift b –polyphen b –custom ClinVar_20200210/clinvar.vcf.gz,ClinVar,vcf,exact,0,CLNSIG,CLNREVSTAT,CLNDN –coding_only’ command. Amino acid substitutions with a SIFT score less than 0.05 were considered potentially functional^24^, and the number of such homozygous non-reference sites was counted per individual. Unmasked and rescaled values are shown as defined above (Fig. 1e). ‘Pathogenic’ ClinVar annotations were also counted (Supplementary Table 1).

### Runs of homozygosity

The number of ROH segments greater than 1 megabase (Mb) and the sum of their length were estimated using bcftools roh^66^ (v.1.11, default parameters) in the NCIG + PNG + high-coverage 1000 Genomes dataset (Fig. 1f and Extended Data Fig. 1b,c) and separately for the SGDP dataset. Given that we are interested in per-individual ROH regardless of recent ancestry, unmasked data were used. Individuals with more than 5% ancestry other than Indigenous ancestry are displayed as dashes in Fig. 1f. For comparison, we show individuals from the SGDP dataset with the most extreme ROH (and their population sample) in Extended Data Fig. 1c.

### Segregating sites and progressive sampling

The number of polymorphic sites observed was calculated as the per-population sample size was progressively increased using the NCIG + PNG (masked) + high-coverage 1000 Genomes dataset. Yarrabah and PNG (Is.) were not included because of variable ancestry other than Indigenous ancestry, and only unrelated individuals with less than 5% ancestry masked were included for the other populations. The count of segregating sites was obtained using the PLINK ‘--freq’ command and custom Unix scripts as the sample size was progressively increased from 1 to 35, taking the average of ten replicates (Fig. 1g).

The level of novel variation observed in a continent, given that all other continents have already been sampled, was estimated for the same dataset with the reintroduction of unrelated individuals from Yarrabah and PNG (Is.) with less than 25% ancestry masked (four individuals from Yarrabah and two from PNG (Is.)). This less-stringent cutoff ensured that a similar number of populations were included from each continent. Populations were pooled into continental groups, and the number of further polymorphic sites observed was scored as the sample was progressively increased from 1 to 80, after first sampling 80 individuals from each of the other five continents, taking the average of ten replicates (Fig. 1g).

### Population structure

Pairwise genetic distances were estimated using the minor allele frequency-corrected covariance (COV)^33,61^ (Extended Data Fig. 2a) calculated using PLINK (v.1.9)^64^; rare allele sharing (Fig. 2d), defined by allele count less than or equal to 5 in the NCIG + PNG (masked, all individuals) dataset; and pairwise outgroup *F*_3_ scores using ADMIXTOOLS (v.5.1, default settings)^36^ (Extended Data Fig. 2b). Ancestry was masked and analysis restricted to sites without missing data in each pairwise comparison; full details are in Supplementary Note 4.

Hierarchical clustering was carried out using the hclust() function of the stats package of R^67^ on the pairwise outgroup *F*_3_ matrix, with relatedness filtering (Fig. 2b).

The ADMIXTURE algorithm^32^ was applied to the NCIG + PNG (masked) dataset with all samples (Extended Data Fig. 3) and after relatedness filtering (Fig. 2c). *K* was varied from 2 to 8, with cross validation supporting *K* = 4 and *K* = 5 (Supplementary Note 4).

The RefinedIBD algorithm (v.102)^68^ was used to infer IBD tract sharing between pairs of individuals in the NCIG + PNG (masked) dataset (Fig. 2d). Variants with a minor allele count of strictly fewer than 8 in the dataset were removed. Default settings were used, including a threshold of 1.5 cM as the minimum IBD segment length. Counts were rescaled to account for the proportion of the genome missing because of masking in each pairwise comparison.

Multidimensional scaling (MDS) was applied to the COV matrix using the cmdscale() function in R (v.5.1) following the approach of ref. ^69^ (Extended Data Fig. 2c).

UMAP (v.0.2.7.0)^70^ was applied as per ref. ^34^ to the top ten components of the MDS output generated from the COV matrix (Fig. 2e).

fineSTRUCTURE (v.4.0.1)^31,33^ was run on unrelated individuals with no discernible ancestry other than Indigenous ancestry from the NCIG + PNG (unmasked) dataset (no individuals from Yarrabah were included because of the requirement for no missing data; Fig. 2f and Extended Data Fig. 4; see Supplementary Note 4 for full details).

To contextualize levels of structure observed among Indigenous Oceanic populations, the hierarchical clustering, ADMIXTURE and RefinedIBD algorithms were applied to other continental cohorts from the 1000 Genomes dataset (Supplementary Note 4).

Pairwise *F*_ST_ was calculated for the Australian and PNG population samples and those of SGDP using the NCIG + PNG (masked) + 1000 G (low-coverage) + SG dataset. *F*_ST_ was calculated using the Eigenstrat software package^61^. To provide an unbiased estimator of *F*_ST_^71^, the dataset was filtered to a subset of sites that were polymorphic in the Mbuti populations of the SGDP collection. The results are shown in Extended Data Fig. 7.

### *F* statistics

*F* statistics were calculated using the NCIG + PNG (masked) + 1000 G (low-coverage) dataset, with further datasets included as described below. ADMIXTOOLS^36^ was used to calculate all *F* statistics, using the Yoruban (YRI) population from the 1000 Genomes as the outgroup, with default parameters, unless otherwise stated.

The degree of shared genetic drift between each Indigenous Australian sample and a panel of Papuan samples was estimated using the statistic *F*_3_(YRI; PNG, NCIG*x*). Here ‘PNG’ is the panel of 25 Highland PNG samples described in ref. ^1^ and ‘NCIG*x*’ represents each Indigenous Australian individual assessed in turn. Significantly higher values of this statistic indicate a population shares more genetic drift with PNG, relative to the other populations (Fig. 3a and Supplementary Note 4).

*F*_4_-statistics of the form *F*_4_^(T)^(YRI, PNG; *X*, *Y*)^72^ were used to infer differing degrees of shared genetic drift between pairs of the Australian populations and PNG. Population nomenclature is as described above, with ‘*X*’ and ‘*Y*’ representing sets of samples from all pairwise combinations of Tiwi, Galiwin’ku, Yarrabah and Titjikala. As is standard^72^, we defined *Z-*scores greater than absolute value 3 to be significant, meaning *Y* shares more drift with PNG than *X* (positive score).

To determine whether populations from South Asia, East Asia or Oceania share the same degree of genetic drift with Titjikala and either Tiwi or Galiwin’ku, *F*_4_-statistics of the form ${F}_{4}^{({\rm{T}})}$ (Asia-*Y*, YRI; Australia-*X*, Titjikala) were calculated on an expanded dataset including the SGDP (Supplementary Note 2), where ‘Asia-*Y*’ is any SGDP sample from South Asia, East Asia or Oceania; and ‘Australia-*X*’ is either the Tiwi or Galiwin’ku sample (Fig. 3b; further details and theoretical justification are given in Supplementary Note 4).

*F*_3_-statistics of the form *F*_3_(AUA*x*; PNG, AUA*y*) were used to assess whether the increased affinity the three northern populations of Australia (Tiwi, Galiwin’ku and Yarrabah) hold with PNG can be attributed to recent Papuan-related admixture. Here ‘PNG’ represents the 25 Highland Papuans, and ‘AUA*x*’ and ‘AUA*y*’ represent one of Tiwi, Galiwin’ku, Titjikala and Yarrabah. There is significant evidence that the population ‘AUA*x*’ has recently received an ancestral contribution from a population related to ‘PNG’ and ‘AUA*y*’ if the statistic is less than −3 (Extended Data Fig. 8c and Supplementary Note 4).

To test whether the additional genetic drift shared between Papuan populations and Tiwi (relative to Titjikala) was uniform across Papuan groups, we incorporated single-nucleotide polymorphism array data from PNG^57^ and compared the outgroup *F*_3_ statistics *F*_3_(YRI; Tiwi, PNG-*X*) to *F*_3_(YRI; Titjikala, PNG-*X*) (Supplementary Notes 2 and 4).

### Demographic modelling of the historical relationships within Australia

We use ABC to assess a range of demographic topologies. Seven plausible topologies were identified and datasets simulated 50,000 times from each with msprime (v.1 within tskit release)^38,73^. The following summary statistics were calculated: *F*_3_ and *F*_4_ statistics, the second and third moments of each *F*_3_ and *F*_4_ statistic, Tajima’s *D*, nucleotide diversity and counts of segregating sites. Statistics were computed directly from tree sequences using the tskit package (development version, since released as v.1.0)^74^. The same set of summary statistics were computed on the NCIG + PNG dataset using ADMIXTOOLS^36^ and PLINK^64^. We checked that the statistics were calculated the same way and return the same values using all software. An ABC–random forest model^75^ was used to infer the most probable scenario and estimate model parameters (Supplementary Note 5).

### Historic autosomal effective population size and isolation

Pairwise IBD tracts were inferred using RefinedIBD (v.102)^76^, and recent effective population sizes were inferred using IBDNe (v.23Apr20.ae9)^41^, with ancestry-specific effective population sizes (ref. ^77^) inferred for Yarrabah and PNG (Is.) using the local ancestry inferred from RFMIX (parameters and sample sizes are detailed in Supplementary Note 6).

Longer-term effective population sizes were inferred with MSMC2 (v.2.1.2)^1,42^ from eight phased haplotypes from four randomly sampled individuals from each population (all autosomes), repeated for five replicates of unique sets of four individuals (some individuals may appear in more than one replicate) and applying masks for mappability, low coverage and ancestry other than Indigenous ancestry (Supplementary Note 6).

Genetic isolation between population pairs was inferred with MSMC2 rCCR using ten replicates of four phased haplotypes per population (two individuals).

### Mitochondrial genetic structure and diversity

Mitochondrial variants were called with GATK (v.3.8-0)^78^ ‘HaplotypeCaller’ with ploidy set to haploid and validated via several metrics including maternal parent–offspring genotype concordance (Supplementary Note 7). Mitochondrial phylogenies were inferred using BEAST (v.2.6.0)^79^, and maximum clade credibility trees were produced with TreeAnnotator^79^. Further Australian and Melanesian mitochondrial sequences were incorporated to better resolve the points of coalescence between lineages (Supplementary Note 7). A dataset of mitochondrial haplogroup frequencies from previous studies was collated to explore the frequencies of haplogroups N13, Q2 and P3 across Australia (Supplementary Note 7).

### Maps

Maps were obtained from Google Maps using the ‘get_googlemap’ function of the ‘ggmap’ package in R^80^, and points were superimposed using ggplot2 (ref. ^81^).

### Reporting summary

Further information on research design is available in the Nature Portfolio Reporting Summary linked to this article.

## Online content

Any methods, additional references, Nature Portfolio reporting summaries, source data, extended data, supplementary information, acknowledgements, peer review information; details of author contributions and competing interests; and statements of data and code availability are available at 10.1038/s41586-023-06831-w.

### Supplementary information

Supplementary Information Supplementary Notes 1–7, including Figs. 1–6, Tables 1–3 and additional references.

Reporting Summary

## Figures and Tables

**Extended Data Table 1:**
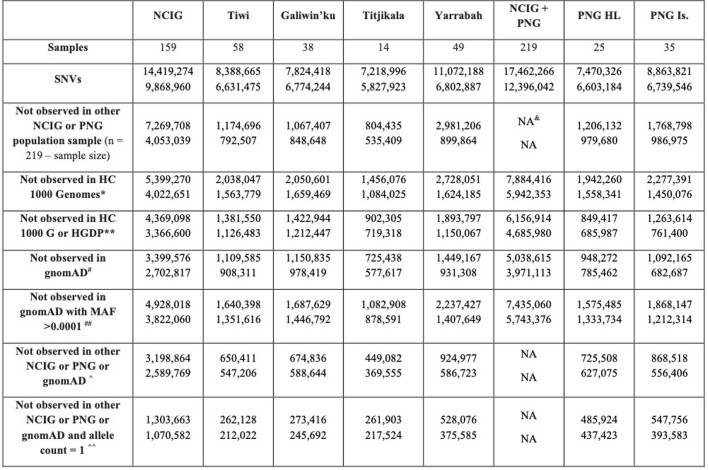
Per population sample count of autosomal SNVs at VQSR = 99.8 before (top) and after (bottom) ancestry masking

^&^Removing all 219 samples leaves zero samples.

^*^High coverage 1000 Genomes from Byrska-Bishop et al., 2022.

^**^1000 G and HGDP from the gnomAD v3.1.2 HGDP + 1KG subset.

^#^gnomAD 3.1.2 (n = 76,156 genome, includes HC 1000 Genomes (n = 2,435) and HGDP (n = 780)).

^##^AF > 0.0001 equates a minimum minor allele count (AC) in gnomAD of 16 assuming a total allele count (AN) of 152,321.

^^^Previously unobserved variation based on these samples.

^^^^Novel Singletons.

## Data Availability

All sequencing data, variant calls and metadata have been deposited in the Australian National Computational Infrastructure, Canberra, under project identifier TE53. Access can be requested by writing to the NCIG Collection Access and Research Advisory Committee, overseen by the Indigenous-majority NCIG Board, at jcsmr.ncig@anu.edu.au. The data are available for general research use subject to meeting the requirements of the NCIG Governance Framework available at https://ncig.anu.edu.au/files/NCIG-Governance-Framework.pdf. Requests for data access for external research will be assessed in accordance with the NCIG Governance Framework.
