# Supplementary material for: Indigenous Australian genomes show deep structure and rich novel variation
Source: Nature. 2023 Dec 13;624(7992):593–601. doi: 10.1038/s41586-023-06831-w (PMC10733150; doi:10.1038/s41586-023-06831-w)
Supplement: Supplementary file 1 — Supplementary Notes 1–7, including Figs. 1–6, Tables 1–3 and additional references. [file 41586_2023_6831_MOESM1_ESM.pdf]

nature portfolio
https://doi.org/10.1038/s41586-023-06831-w

Supplementary information

Indigenous Australian genomes show deep structure and rich novel variation

In the format provided by the authors and unedited

Supplementary Information Guide

Supplementary Note 1: Samples, Ethics and Community Consultation..1

Supplementary Note 2: Sample Processing, Phasing and Ancestry Analysis 3

Supplementary Note 3, Global Variant Sharing...6

Supplementary Note 4: Population Structure and F statistics..9

Supplementary Note 5: Demographic modelling of the historical relationships within Australia........... 13

Supplementary Note 6: Historic Autosomal Effective Population Size and Isolation............................. 21

Supplementary Note 7: Mitochondrial Genetic Structure and Diversity 24

Supplementary References.. 25

Supplementary Note 1: Samples, Ethics and Community Consultation

Nature of the original collection

The Indigenous Australian data analysed here is part of the collection of biological samples, genomic data and documents managed by the National Centre for Indigenous Genomics (NCIG), at the John Curtin School of Medical Research (JCSMR) at the Australian National University (ANU).

The collection was created as part of the biomedical research carried out at JCSMR by Robert Kirk and others, beginning in the 1960s, and includes biological samples and additional research records obtained from approximately 7,000 people from at least 40 collection sites, from Indigenous communities in western and northern Australia.

Governance & Ethical Oversight

In 2012 an external consultative committee of leading Aboriginal and Torres Strait Islander Australians recommended that the ANU develop a managed collection overseen by an Indigenous-majority Board who manage the collected samples and records. NCIG, a statutory body within ANU, was founded in 2013. Governance is bound by the National Centre for Indigenous Genomics Statute (2016, updated 2021)[1]. This Federal statute requires a majority of Aboriginal and Torres Strait Islander representatives on the NCIG Board, ensuring Indigenous oversight of the Centre's decision-making processes and activities. The Board is the custodian of the NCIG Collection and has established Governance and Ethical Oversight Frameworks.

NCIG develops protocols and provides ethical oversight for the collection to be used by researchers and clinicians to the benefit of the people who have donated samples to the collection, their communities and descendants, the broader Indigenous community, and the general Australian community. NCIG provides a framework for appropriate and respectful Indigenous engagement in genome research in Australia. The NCIG Board ensures a capacity to innovate, to develop new standards of ethical practice that go beyond current compliance requirements, and to improve processes over time considering operating experience and in response to community expectations.

Engagement & Outreach

The NCIG engagement practices respect the principles and values of Aboriginal and Torres Strait Islander cultures. These practices inform Indigenous Australians of the existence of a collection of biological samples (blood and derived products) collected from Indigenous communities between the 1960s and 1990s. They

explain the potential use of the samples to create important new medical knowledge which will specifically address Indigenous health issues. They further explain other potential uses of the collection which may benefit Indigenous Australians and seek consent to sequence the samples to create an Indigenous genome database for research.

For this project, NCIG engaged with the Traditional Owners, Community Elders, and other appropriate community representatives to inform the community about the research. This involved: contact with the Shire Service Manager/s; inquiries with community stakeholders as required; arranging interpreters; promoting the visit in advance; and preparing outreach material including plain English project summaries and consent forms. We worked directly with the following organisations:

1. Yarrabah, Far North Queensland: Yarrabah Shire Council,
2. Galiwin'ku, East Arnhem Land, Northern Territory: Yalu Aboriginal Cooperation
3. Titjikala, Mary Vale Station, Central Australia: Titjikala Health Centre
4. Tiwi Islands, Northern Territory: Tiwi Land Council

Initial work by NCIG focussed on informing communities about the existence of the historical collection and on seeking advice on its continued maintenance and possible future use. NCIG further began the process of seeking permission from the relevant individuals and communities to sequence some of the existing biological samples. During this process NCIG sought, and received with consent (see below), new samples of blood or saliva from current members of the communities we engaged with (some, but not all of whom were part of the historical collection). It is these new samples that form the basis of the dataset analysed herein.

Informed Consent and sample collection

Via a community liaison officer, official translation services, local community translators and a video animation, confidentiality agreements, project information and consent forms were communicated to local community organisations, community leaders and participants. Questions were answered and participant concerns addressed in English and using translators. Individuals fell into three classes; 1) Individuals that had provided a blood sample *circa* 2012 as part of a study of chronic kidney disease in the Tiwi Island community[2] and provided new informed consent for this study 2) Individuals with a sample in the historic NCIG collection but nonetheless provided a new saliva sample, and 3) Individuals with no prior connection to the NCIG collection that chose to participate during community engagement and provided a saliva sample. All individuals provided informed personal consent during community visits between *circa* 2015 and 2018. Additionally, one individual from each community (two from Tiwi; five in total) provided a blood sample that was used to extract high molecular weight DNA for long-read sequencing.

Return of Results

The results contained in this paper were returned to communities and participants in several ways. While the data used for the scientific research was anonymised, NCIG maintained the contact details for all participants and thus was able to recontact people. A plain language summary of the final draft of this manuscript was produced. This was sent to the community partners in each community. The NCIG Community Liaison Officer ran a workshop to return results in two of the four communities. Due to logistical difficulties the workshop was unable to be held in the other two communities, although it is planned to run the workshop in these communities in the future. The plain language summary was provided to each participant, either in person or via mail or email. The community liaison officer was available to take questions, either in person during the workshop, or over the telephone. The draft of this paper was also available to those who wanted it. Many participants took advantage of the opportunities for more information offered. Participants were overwhelmingly satisfied with the outcomes of the study and the process for returning results.

Supplementary Note 2: Sample Processing, Phasing and Ancestry Analysis

An overview of the processes used to produce the datasets analysed herein is given in the *Methods*. Here we give further details.

External datasets

Fastq files of high-coverage whole-genome sequencing of 25 individuals from Highland Papua New Guinea[3] were obtained with permission from the European Genome-phenome Archive (EGA); dataset EGAD00001001634. Fastq files of high-coverage whole-genome sequencing of 35 individuals sampled from the Bismarck Archipelago of Northern Island Melanesia, Papua New Guinea, Accession PRJNA314367, were obtained with permission from the authors[4]. Both datasets were processed jointly with NCIG samples.

VCF files for the SGDP[5], the LC 1000 Genomes[6] and the HC 1000 Genomes[7] are publicly available. Illumina Infinium Multi-Ethnic Global array genotypes from 378 individuals from PNG[8] were obtained with permission from EGA; dataset EGAD00010001326.

Read mapping and variant calling

Reads from the NCIG and Papuan samples were mapped with bwa mem (v0.7.17)[9] to GRCh38 including alt-contigs obtained from the GATK resource bundle. Files were converted to BAM format and indexed with samtools (v1.9)[10]. Where necessary, multiple BAM files were merged with biobambam2 bammerge (v.2.0.95)[11]. Files were sorted and duplicates marked with biobambam2 bamsormadup.

Joint variant calling was carried out with GATK (v.3.8-0)[12] over recommended genomic intervals following standard practice and default settings, except as stated below. Base Quality Score Recalibration (BQSR) was carried out with BaseRecalibrator with recommended knownSites, bqsrBAQGapOpenPenalty=30.0 and allowPotentiallyMisencodedQuals. Per sample gVCF files were generated with HaplotypeCaller --emitRefConfidence GVCF -G Standard -G AS_Standard --BQSR sampleID.recal.table --GVCFGQBands 10,20,30,60. BAM files were converted to CRAM format and indexed with samtools. Multi-sample VCFs were generated with GenotypeGVCFs. Variant Quality Score Recalibration (VQSR) was run for SNPs and INDELs with VariantRecalibrator with tranches set to 100.0, 99.9, 99.0, 90.0, followed by ApplyRecalibration with ts_filter_level=99.0. Variants were annotated based on dbsnp_146 with VariantAnnotator, generating a multi-sample VCF for the autosomes, chrX, chrY (Mitochondria were considered separately, see Supplementary Note 6).

VQSR was separately run at ts_filter_level=99.8 to allow comparison with the high coverage 1000 Genomes dataset[7].

Merging datasets

The following combinations of dataset were produced:

- NCIG + PNG (masked) intersected with the 1000 Genomes low coverage (LC) [6]; referred to as 'NCIG + PNG (masked) + 1000G (LC)'.
- NCIG + PNG (masked) intersected with the Simons Genomes Diversity Panel (SGDP)[5] and the LC 1000 Genomes; referred to as 'NCIG + PNG (masked) + 1000G (LC) + SG'.
- NCIG + PNG (masked) intersected with the LC 1000 Genomes and SNP array genotypes 379 individuals from 85 language groups in Papua New Guinea [8]; referred to as 'NCIG + PNG (masked) + 1000G (LC) + PNG_SNP_CHIP'.
- NCIG + PNG (masked) merged (union) with the 1000 Genomes High Coverage (HC) [7]; referred to as 'NCIG + PNG (masked) + 1000G (HC)'.
- NCIG + PNG (no ancestry mask) merged (union) with the 1000 Genomes High Coverage (HC); referred to as 'NCIG + PNG + 1000G (HC)'.

The intersection of datasets was generated using the bcftools 'isec' command, before combining them with the bcftools 'merge' command and retaining only biallelic SNVs. Only sites identified as having a non-reference allele in both datasets are retained via this process.

The union of datasets were generated using the plink '--bmerge' command with uncalled sites set to homozygous reference.

Comparison of phasing methods

Many of the methods we apply to the NCIG data require haplotype-phased genotypes. We ultimately phased using ShapeIT (v2.12)[13], using both the LC 1000 Genomes reference panel and phase informative reads but we tried various methods and compared them to obtain the best possible phasing of the data. We measured rates of switch error by comparing Illumina WGS data, phased using ShapeIT (v2.12), to data phased using 10X Genomics 'Chromium' platform for five samples; two from Tiwi, one each from the other three communities. We observed the lowest switch error estimates, typically lower than 1-2%, when phasing using the 1000 Genomes reference panel and phase informative reads. Particularly low switch error estimates were observed for the Tiwi samples. Details available on request.

Switch error rates in Indigenous and non-Indigenous regions of the genome

For the Yarrabah 10X sample, which was inferred to have ~25% non-Indigenous ancestry, we compared rates of switch error in regions of the genome inferred to be homozygous and heterozygous for Indigenous ancestry. We found phasing using the 1000 Genomes reference panel to result in lower switch error rates in regions of heterozygous European/Indigenous ancestry than in regions where both homologous chromosomes are inferred to be of Indigenous ancestry (Mann Whitney U test $p < 10^{-16}$). When phasing "internally" without a reference panel, switch error rates were lower in regions where both homologous chromosomes are inferred to be of Indigenous ancestry than in regions heterozygous for Indigenous/European ancestry (Mann Whitney U test $p < 10^{-16}$). Details available on request.

ADMIXTURE inferred non-Indigenous ancestry proportions

Non-Indigenous ancestry proportions were inferred using ADMIXTURE [14] (*Extended Data Figure* 1A) and verified (see below) using F_4-ratios, PCA (data not shown) and RFMIX. When running ADMIXTURE, we found 'K' values (the number of clusters) of '6' and '7' to result in the lowest cross-validation scores. Running ADMIXTURE at K=7 returned a slightly lower cross validation score than K=6, inferring a split of the 'Oceania' specific cluster into two separate clusters: one highest in Tiwi Island samples, and another highest in the PNG Highland samples. These two components are present only in populations from 'Oceania' (NCIG + PNG dataset samples) and, importantly, do not result in noticeable changes in the inferred non-Indigenous ADMIXTURE proportions.

F_4-ratios for inferring non-Indigenous ancestry proportions

To estimate European ancestry proportions using F_4-ratios, the following F_4-ratio was computed:

$$alpha(EUR) = \frac{F4(CEU, YRI; X, PNG)}{F4(CEU, YRI; GBR, PNG)}$$

In which 'PNG' represents a panel of the 25 highland Papuan individuals described in the study of Malaspinas et al. (2016)[3], 'X' represents the individual from the NCIG + PNG dataset who is to be assessed for non-Indigenous ancestry, and YRI, CEU and GBR represent the 1000 Genomes populations 'Yoruba', 'European Caucasians from Utah', and 'Great Britain', respectively. This F_4-ratio was only computed on samples inferred using ADMIXTURE to have less than 7.5% non-Indigenous ancestry from another source (i.e., East Asian). We found European global ancestry proportions inferred using the F_4-ratio to be strongly correlated with those inferred using ADMIXTURE (details available on request).

East Asian global ancestry was assessed using the follow F_4-ratio, again on samples with less than 7.5% non-Indigenous ancestry from another source:

$$alpha(EAS) = \frac{F4(CHB, YRI; X, PNG)}{F4(CHB, YRI; CHS, PNG)}$$

In the above, 'CHB' and 'CHS' denote the 1000 Genomes populations 'Chinese Han in Beijing' and 'Han Chinese South' respectively. East Asian global ancestry estimates computed using the F_4-ratio were strongly correlated with those inferred using ADMIXTURE. Results of all F_4-ratio analyses are available from the authors on request.

<u>RFMIX inferred non-Indigenous ancestry proportions</u>

We calculated RFMIX[15] inferred non-Indigenous ancestry proportions, by summing the length of tracts inferred to belong to each ancestry type for each sample. We found these measures to be strongly correlated with ADMIXTURE inferred global ancestry estimates (K=6) for European and East Asian ancestries (Spearman's Correlation Coefficient = 0.999 when comparing European global ancestry estimates; and 0.996 when comparing East Asian global ancestry proportions).

<u>RFMIX parameter values and reference panel composition</u>

For the RFMIX inference, we assembled a reference panel including a broad range of non-Indigenous source populations. This consisted of 30 African samples: 10 from each of YRI, MSL and ESN, 30 European samples: 10 from each of TSI, GBR and FIN, 30 East Asian samples: 10 from each of CHB, CHS, KHV and 30 South Asian samples: 10 from each of GIH, STU, ITU. 30 unrelated individuals from the NCIG + PNG (unmasked) dataset were selected based on ADMIXTURE, PCA and F_4-ratio analyses that show they have minimal non-Indigenous ancestry. 15 Papuan individuals from the study of Malaspinas et al. (2016)[3] were included in the reference panel, with three individuals being randomly selected from each of the 5 populations.

RFMIX v1.5.4 was run in 'PopPhased' mode using the 'phase correction' feature, and a recombination map generated using the 'armartin' local ancestry pipeline[16]. All remaining RFMIX parameters were set to their default values, aside from a node size of 5, as per prior studies[17,18].

<u>Tiwi samples inferred to derive Indigenous ancestry from communities other than Tiwi</u>

It became apparent from a number of population structure analyses (see Supplementary Note 4 for technical details of each), that a small subset of Tiwi individuals had different genomic characteristics (see below) to members of the general Tiwi population. There were ten such samples in the complete data, although two of these were removed in the kinship analysis. To avoid confounding key inferences, these individuals were removed from downstream analyses of population structure and demography.

When performing MDS, these samples formed a separate cluster between the remaining Tiwi samples, and those from other Indigenous communities. ADMIXTURE revealed that these samples had substantial inferred ancestry proportions of components predominant in Galiwin'ku, Yarrabah and Titjikala. A heatmap showing pairwise COV distance measures showed these samples have a lessened affinity for the general Tiwi population, as well as to one another.

Collectively, these results imply these individuals have Indigenous Australian ancestry that is derived, either partially or fully, from subpopulations likely to be of non-Tiwi origin. This hypothesis is supported by the finding that these individuals have highly elevated measures of nucleotide diversity, which is a known feature of individuals who derive ancestry from highly differentiated sub-populations[19], and decreased ROH (*Extended Data Figure 1C*). Further details of this analysis are available upon request.

Supplementary Note 3, Global Variant Sharing

As described in the Main Text, we explored patterns of variant sharing between Indigenous Australian groups and the worldwide populations represented in the Thousand Genomes dataset. This was to improve to our understanding of the suitability of available reference resources and databases for future studies of Indigenous Australian genomics, health and disease. These analyses considered genome-wide variation in population samples of various sizes (Fig. 1A-B, Supplementary Figure 1A-E), and when considering variation that is expected to be relevant for genetic disease (Supplementary Figure 1F and Supplementary Table 1). Full details of the analytical approaches are given in the Brief Methods (see 'Genomic variation').

Variation in genes associated with Type 2 Diabetes

As our cohort lacks phenotypes, associating genetic variation with diseases relevant to Indigenous communities is very difficult, although we make some simple observations here. As an example, we consider coding variation in 32 genes associated with type 2 diabetes (T2D)[20] (see Brief Methods), a disease with high prevalence in Indigenous Australians. Considering equal size (5) samples for each population sample in our data (including PNG) and the non-African 1000 Genomes samples, we consider non-synonymous variants in these T2D genes. For non-African 1000 Genomes populations, the range of total non-synonymous variants in T2D genes is 71 (FIN) to 102 (PUR), whereas for our Oceania population samples the range is 32 (PNG Is.) to 62 (Titjikala & Tiwi). Restricting to variants that either population private, or private to continent we observe 2-4 (median 4) non-synonymous variants for East Asian populations, 5-8 (median 6) for South Asia, 1-5 (median 4) for Europe, 1-4 (median 3) for the Americas, and 1-5 (median 3.5) for Oceania (noting that ancestry masking affects the estimates for PNG (Is.) and Yarrabah which are the only values below 3). This is consistent with genome wide patterns from Figure 1; Oceanic populations have fewer variants than other populations, yet similar numbers of population & continent private variants. Overall, we see no elevation of non-synonymous variants in T2D genes in Indigenous Australians compared to elsewhere in the world.

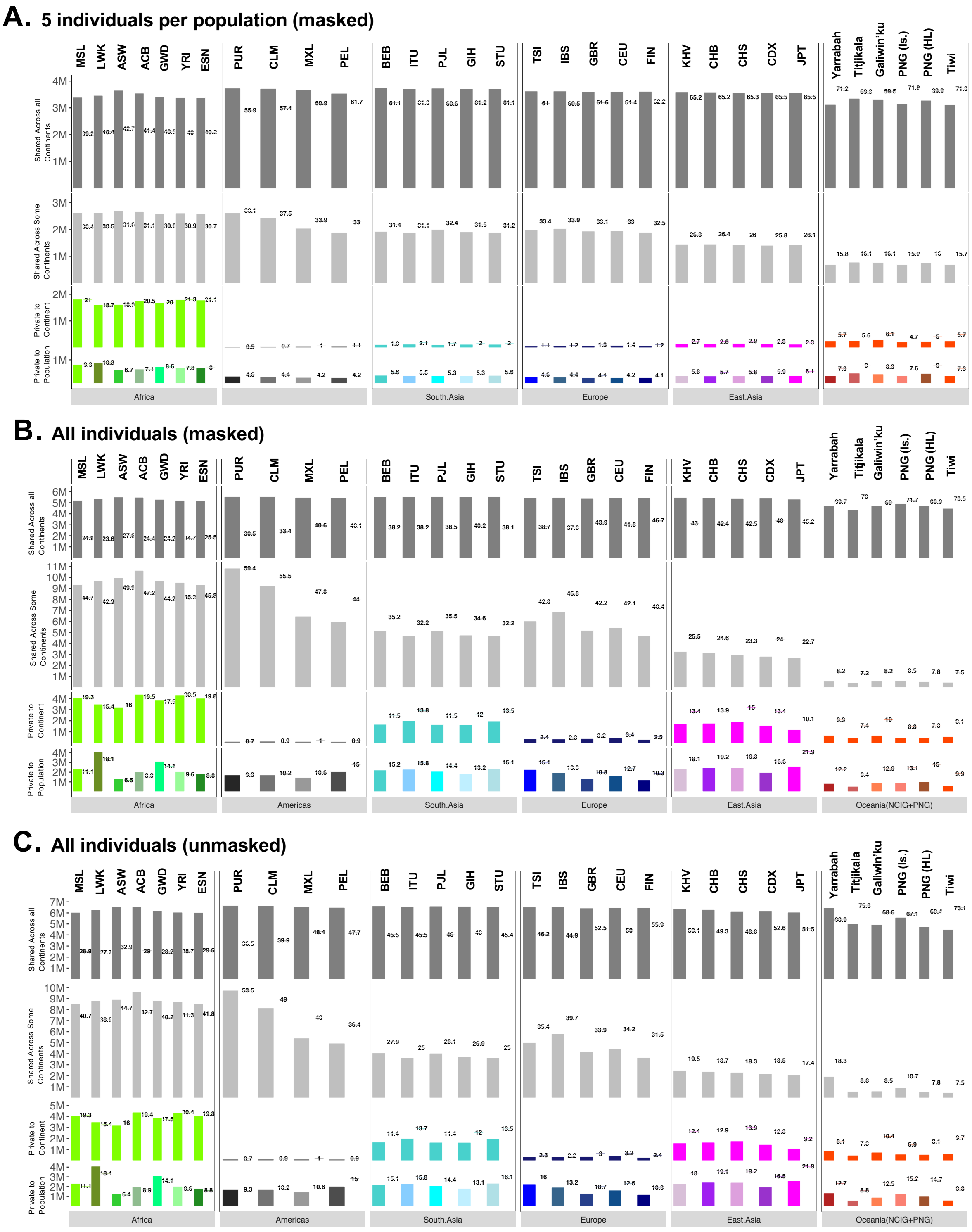

A. 5 individuals per population (masked)
B. All individuals (masked)
C. All individuals (unmasked)
MSL
LWK
ASW
ACB
GWD
YRI
ESN
PUR
CLM
MXL
PEL
BEB
ITU
PJL
GIH
STU
TSI
IBS
GBR
CEU
FIN
KHV
CHB
CHS
CDX
JPT
Yarrabah
Titjikala
Galiwin'ku
PNG (Is.)
PNG (HL)
Tiwi
Shared Across all Continents
Shared Across Some Continents
Private to Continent
Private to Population
Africa
Americas
South.Asia
Europe
East.Asia
Oceania(NCIG+PNG)

D. 15 individuals per population (masked)

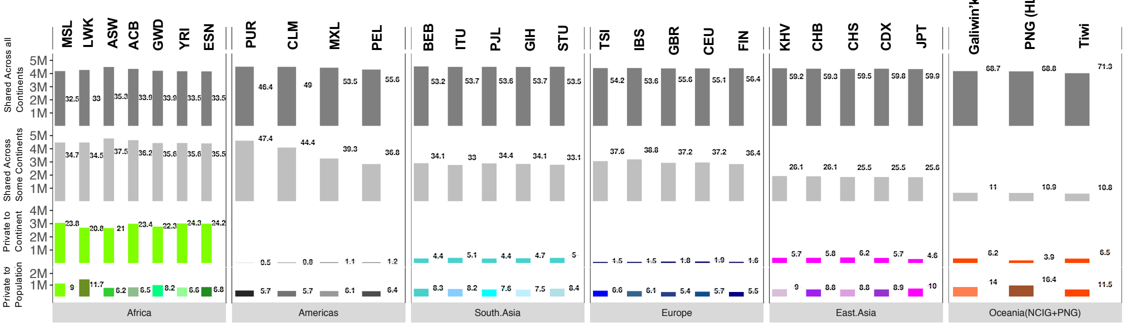

E. 25 individuals per population (masked)

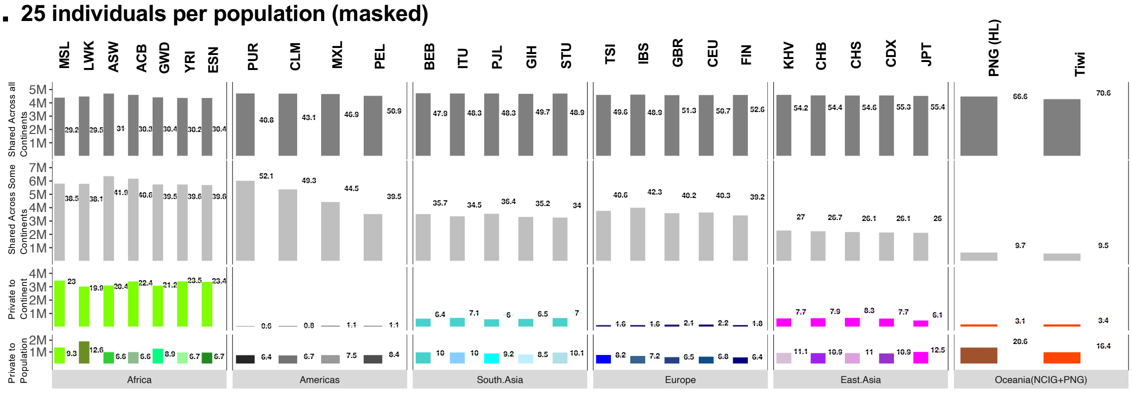

F. 5 individuals per population (masked) – only ClinVar pathogenic

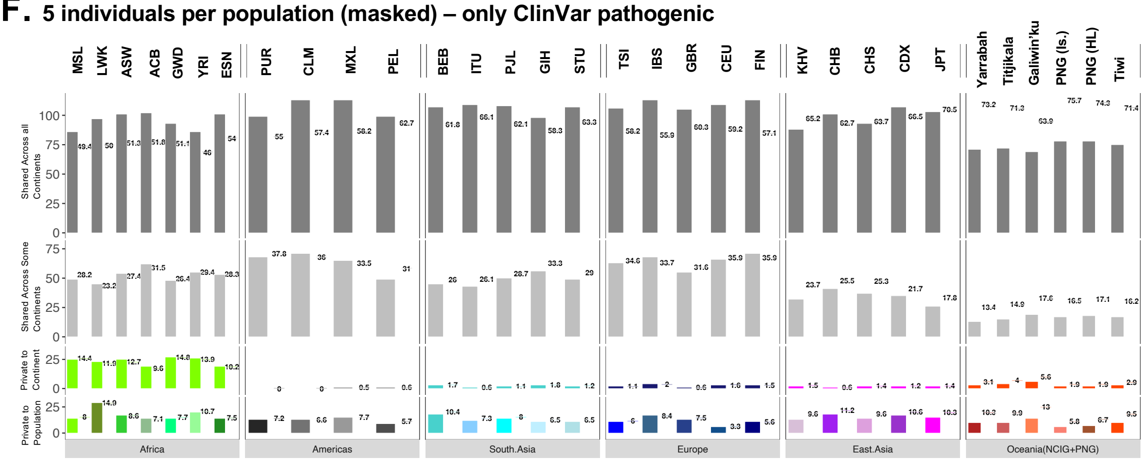

Supplementary Figure 1. Variant sharing across populations and continents for the NCIG, PNG and HC 1000 Genomes dataset. **A.** The subsample of 5 individuals per population (as per Fig.1). **B.** All individuals after ancestry masking in the Oceania samples. **C.** All individuals with no ancestry masking. **D.** A subsample of 15 individuals per population (where available). **E.** A subsample of 25 individuals per population. **F.** The subsample of 5 individuals per population restricted to only variants classified as “Likely pathogenic” or “Pathogenic” in ClinVar. Per population sample counts (bars) and proportions (numbers; value depicted by bar at midpoint of number) of biallelic single nucleotide variants that are: private to a population sample; private to a continent (shared between population samples within that continent; but not found in any of the other five continents included); shared across continents (found in multiple continents, but not all six); and shared across all six continents.

Supplementary Table 1. Mean number of predicted deleterious SNVs across high coverage 1000 Genomes, NCIG and PNG samples.

	African	Admixed American	South Asian	European	East Asian	Oceania (NCIG+PNG)	Galiwinku	Titjikala	Tiwi	Yarrabah
SIFT Deleterious ^	2765	2286	2294	2238	2259	2546	2544	2564	2484	2635
PolyPhen Probably Damaging ^	1027	849	860	834	836	962	960	985	927	992
ClinVar "Pathogenic" Total (Homozygous)	4 (1)	5 (1)	5 (1)	5 (1)	5 (2)	4 (1)	4 (1)	4 (1)	3 (2)	4 (1)

^ Sum of sites in heterozygous and homozygous state.

Supplementary Note 4: Population Structure and F statistics

Contextualising Indigenous Australian Population Structure: Comparison with 1000 genomes continental cohorts

To provide context for the findings from the Indigenous Australian sample we ran a parallel analysis on four continents of the LC 1000 Genomes dataset (Africa, Europe, East Asia, and South Asia). Although many features of these continental groups are different from those of Oceanic populations (e.g., demographic history, population sizes, etc.), they are roughly comparable on the basis of geographic scale, and provide some context for the levels of structure inferred within the NCIG + PNG dataset. Hierarchical clustering was implemented as described below for the NCIG + PNG (masked) dataset (excluding related individuals; sample size = 141) on randomly chosen subsets 140 samples from each of Europe, East Asia, and South Asia (28 individuals per sub-population; sample locations shown in *Extended Data Figure 6*). Due to the need of an African outgroup population for this statistic, Hierarchical clustering wasn't performed for the continent of Africa.

ADMIXTURE was also run as described below on the same subset of samples from each continent. This analysis was also run on a subset of 196 African samples (28 individuals from each of the 7 African sub-populations). The results of these analyses are shown in *Extended Data Figure 6* and are presented in the same format as the NCIG + PNG (masked) dataset, which features in Main text Fig. 2 (panels A, B and C).

Pairwise Genetic Distances

Two measures of pairwise genetic distance were calculated on the *NCIG+PNG (masked)* dataset: the *minor allele frequency corrected covariance* (COV)[21,22]; and pairwise outgroup F_3 scores. The COV distance measures were calculated using PLINK software[23], and outgroup F_3 distances were calculated using the ADMIXTOOLS software package[24] (default settings), with Yoruba as the root population. For the latter, outgroup F_3, analysis, the NCIG + PNG (masked) + 1000G (LC) dataset was used, which includes Yoruba.

As samples from the dataset have variable degrees of missing data due to ancestry masking, only loci for which both samples are not missing data were used to calculate the pairwise distance. This approach follows that of Browning *et al.* (2016)[25]. We produced each of these distance matrices both before and after relatedness-based filtering. For the purposes of visualising patterns of similarity between populations, we show versions of these matrices (in the form of heatmaps) which include all samples in *Extended Data Figures 2A* and *B*. We perform the downstream population structure analyses on matrices which have been filtered to remove up to and including second degree relatives.

Hierarchical Clustering

The Hierarchical clustering dendrogram was generated from the outgroup F_3 statistic matrix described above, with relatedness filtering (Fig. 2B). To cluster we use the R function hclust()[26] (with all parameters set to default, aside from 'method = "ward.D2"') and the outgroup F_3 statistic matrix.

ADMIXTURE

In initial analyses we applied the algorithm ADMIXTURE[14] to the *NCIG+PNG* dataset merged with the LC 1000 Genomes to identify individuals with evidence of non-Indigenous ancestry (see Supplementary Note 2). Here we apply the ADMIXTURE algorithm to the *NCIG+PNG (masked)* dataset to explore structure between the Indigenous Australian and Papuan populations.

Similar to the approach of Conley et al. (2017)[18], we ran the ADMIXTURE algorithm on an ancestry-masked dataset, which contains variable amounts of missingness for each individual. The algorithm was run with the specified number of clusters, K, from K=2 to K=8 inclusive. The lowest cross validation scores (i.e., the best supported models) were for K=4 (0.21201) and K=5 (0.21296), although there is very little difference in the scores across all values of K. We note that higher values of K result in partitions closely matching the population labels. ADMIXTURE was run both before (*Extended Data Figure 3*) and after filtering out related individuals (Fig. 2C; for K= 7).

Rare Allele Sharing

To explore the strength of rare allele sharing within and between Indigenous communities we first determined the rare alleles carried by each individual. Here we define an allele as rare if it has a count less than or equal to 5 in the *NCIG + PNG (masked)* dataset. Note this is done on the full dataset including related individuals (who are expected to share more rare alleles). For each pair of individuals, we count the number of rare alleles they share and then correct for the proportion of the genomes which were missing in the pairwise comparison to produce a pseudo-count of the number expected across the entire genome. The results are shown in Fig. 2D.

Pairwise Identity by Descent

The RefinedIBD algorithm[27] was used to quantify the degree of Identical by Descent (IBD) tract sharing between pairs of individuals in the *NCIG+PNG (masked)* dataset. Following the recommendations of the RefinedIBD developers, we removed all variants from the *NCIG+PNG (masked)* variant call file (VCF) with a minor allele count of strictly less than 8 in the dataset.

The default settings for the algorithm were used, including a threshold of 1.5 centimorgans as the minimum length of a shared IBD segment, which relates to very recent sharing of IBD tracts, largely within the past several tens of generations[28] (this is probably inappropriate for the situation we are investigating and demonstrates the need for caution when applying standard statistical genetics algorithms to populations like those of Australia).

RefinedIBD was applied to the full dataset including related individuals (who are expected to share more tracts IBD). Pairwise counts of the number of IBD tracts shared by each pair of individuals were then produced using custom Unix scripts, and counts were rescaled to account for the proportion of missingness (due to ancestry masking) in each pairwise comparison. The results are shown in Fig. 2D.

To attain a qualitative comparison of the extent of IBD sharing within and between Indigenous Australian populations and to give these results context, RefinedIBD was also applied to the African, European, East Asian, and South Asian cohorts of the TGP. These cohorts are roughly comparable to the NCIG + PNG dataset on the basis of geographic distance, although crucially the populations have relatively large census and effective population sizes, meaning one expects far fewer tracts to be recently shared IBD between individuals (recall the default settings for RefinedIBD mean that only recent shared IBD is investigated). We recognise that the population dynamics and demographic history of these cohorts are likely very different to these Australian

Indigenous communities, but we merely intend this analysis to provide the reader with context when examining the results for the NCIG data.

The phased low coverage (LC) VCF file supplied with the 1000 Genomes data was restricted to samples from each continental ancestry group (Africa, Europe, East Asia, and South Asia), before removal of all variants with minor allele count of strictly less than 8. RefinedIBD was run independently on each of these four continental datasets, with identical, default parameters to the analysis on the NCIG+PNG (masked) dataset. Counts of the number of tracts shared within each pairwise comparison of individuals were recorded in the same manner (*Extended Data Figure 5*).

Multidimensional Scaling

To generate a low dimensional representation of the COV matrix described above, multidimensional scaling (MDS) was applied using the cmdscale() function in R. This approach follows very closely that of Browning *et al.* (2016)[25], except that the distance measures used relate to diploid, as opposed to haploid genotypes. While MDS was applied to matrices derived from the *NCIG + PNG (masked)* dataset both before and after applying relatedness-based filtering, we only show the results after filtering (*Extended Data Figure 2C*).

Uniform Manifold Approximation and Projection

UMAP[29] was applied to the top 10 components of the MDS output generated from the COV matrix obtained from the NCIG+PNG (masked) dataset, after filtering to unrelated individuals.

fineSTRUCTURE

To investigate population structure on potentially very fine scales we ran the fineSTRUCTURE algorithm[21,30]. A condition of running fineSTRUCTURE is that all individuals must be typed at all loci considered (i.e., there is no missing data). Consequently, for this analysis we removed individuals from the *NCIG+PNG (unmasked)* dataset that had any discernible non-Indigenous ancestry, thus removing all samples from Yarrabah. Furthermore, due to the sensitivity of the algorithm to relatedness-based haplotype sharing, all individuals with any detectable degree of relatedness were excluded from this analysis. This resulted in 78 samples: Tiwi-Bathurst 19, Tiwi-Melville 15, Galiwin'ku 13, Titjikala 6, PNG (HL) 25.

To run fineSTRUCTURE, variant files were converted into CHROMOPAINTER format using scripts native to the fineSTRUCTURE package. This included an interpolated recombination map file, which was produced using the 'convertrecfile.pl' script using the HapMap GRCh38 recombination map as a template[31]. CHROMOPAINTER was run with default settings, and per-chromosome co-ancestry matrices were combined using 'chromocombine'. The fineSTRUCTURE clustering algorithm was run for 1,000,000 iterations on the 'chunk counts' co-ancestry matrix, with a burn-in period of 1,000,000 iterations; trees were sampled every 1000 iterations.

A hierarchical clustering tree was constructed from the partitions identified in the MCMC which attained the maximum *a posteriori* value (K=23). This tree is shown (up to 13 clusters) in *Extended Data Figure 4A*. To demonstrate the concordance of geography with the genetic population structure inferred by fineSTRUCTURE, *Extended Data Figure 4B* shows a map with all samples positioned at their sampling location and coloured by their cluster assignment when there are five clusters.

The pairwise coincidence matrix generated from the fineSTRUCTURE MCMC, recording the proportion of sampled iterations that any pair of individuals appear in the same cluster, is visualised in *Extended Data Figure 4C*. A second hierarchical clustering tree was produced from the maximum concordance state inferred by fineSTRUCTURE after running an additional 100,000 hill climbing iterations. The topology of this tree was identical to the maximum *a posteriori* state tree depicted in *Extended Data Figure 4A*. Multiple independent replicates of this tree building process demonstrated the robustness of the population partitioning and tree topology (data not shown).

Outgroup F_3-statistics of the form F_3(YRI; PNG, NCIGx)

We calculated outgroup F_3-statistics of the form F_3(YRI; PNG, NCIGx) to assess whether Indigenous Australian populations each shared the same amount of genetic drift with PNG (Fig. 3A). To test for statistically significant differences in the magnitude of this statistic between Australian populations, we used the approach of Malaspinas et al. (2016)[3]. This involved applying a Kruskal Wallis test, implemented using the Kruskal.test() function of the stats package in R, to test whether the grouped (by sample location) data are derived from the same distribution.

We also applied a pairwise Mann Whitney U test, implemented in the pairwise.wilcoxon.test() function of the stats package in R (with the paired=FALSE option), to test whether samples from one population are likely to have a higher F_3-statistic value than the other (that is the samples come from different distributions). A Bonferroni correction was applied to account for multiple comparisons (*Extended Data Figure 8A*).

We also tested whether the magnitude of this F_3-statistic value for samples from Yarrabah was significantly correlated with the proportion of recent Papuan related ancestry (calculated post-masking) in that sample inferred using RFMIX. To do this, we calculated the Spearman's correlation coefficient, and generated a p-value using the 'AS 89' algorithm implemented in the cor.test() function of the stats package in R.

F_4 statistics of the form $F_4^{(T)}$(Asia-Y, YRI; Australia-X, Titjikala)

The F_3-statistic analyses described in the preceding section point to a non-cladistic relationship of Australian populations with respect to those of Papua New Guinea. As noted in the Main Text, this finding could be explained by several demographic scenarios. Three plausible scenarios include:

1. Genetic interaction between the ancestral populations of PNG and Northern Australia, while the two landmasses were still connected (i.e., ancient population structure).

2. Common admixture from an external source population (e.g., from Island Southeast Asia) into both PNG and the populations of Northern Australia.

3. Recent admixture from a PNG-related source population into the populations of Northern Australia (e.g., 'Makassar' individuals from Sulawesi).

To differentiate between these three scenarios, data from the SGDP, which incorporates a wide sampling of individuals from Southeast Asia, were included.

As described in the Brief Methods, we calculated F_4-statistics of the form $F_4^{(T)}$(Asia-Y, YRI; Australia-X, Titjikala) for a wide selection of Asian and Oceanic populations from the SGDP. We interpreted a Z-score more than three standard deviations from zero as sufficient evidence to reject the proposed tree topology and conclude that the SGDP population in question shares more genetic drift with Tiwi or Galiwin'ku than it does with Titjikala (Fig. 3B). Since none of the Asian samples has a significant Z-score (while the two PNG samples do) we can exclude Scenario 2.

Note, we follow Peter et al. (2016)[32] with the superscript (T) denoting the use of the F_4-statistic as a test statistic, rather than a measure of internal branch length.

F_3-statistics of the form F_3(AUAx;PNG,AUAy)

To test the third of the plausible scenarios listed above explaining the non-cladistic relationship of the Australian samples to PNG (i.e. that there was recent admixture from a PNG related source population into the populations of Northern Australia), we computed outgroup-F_3 statistics of the form F_3(AUAx;PNG,AUAy). Using the theory of Patterson et al. (2012), a Z-score of less than -3 indicates statistically significant evidence of admixture [24] (*Extended Data Figure 8C*).

Of all pairwise comparisons of Australian populations, the only two which yielded significant values were F_3(Tiwi_Outlier;PNG,Bathurst) and F_3(Tiwi_Outlier;PNG,Melville), in which 'Bathurst' and 'Melville' represent samples from each of the two Tiwi Islands, and 'Tiwi_Outlier' represents the subset of Tiwi samples exhibiting atypical clustering in the population structure analyses (described above). We noted that one sample from this 'Tiwi Outlier' group was inferred to have a high proportion of Papuan related ancestry using RFMIX, which may explain this signal.

Outgroup F_3 Statistics of the Form F_3(YRI; Tiwi, PNG-X) and F_3(YRI; Titjikala, PNG-X)

To assess whether the increased degree of genetic drift shared with Tiwi relative to Titjikala is uniform across Papuan groups, we compared the outgroup F_3 statistics F_3(YRI; Tiwi, PNG-X) and F_3(YRI; Titjikala, PNG-X). Here, 'PNG-X' is a PNG individual from the NCIG + PNG (masked) + 1000G (LC) + PNG_SNP_CHIP dataset, and 'Tiwi' and 'Titjikala' represent the entire samples from Tiwi and Titjikala.

We produced a scatterplot of these two F_3-statistic values and plotted below each point an estimate of Papuan global ancestry inferred using the ADMIXTURE algorithm at K=2, using an identical approach to the original study[8].

The scatterplot in *Extended Data Figure 9B* shows that points are tightly clustered around a trend line. We note that if the increased shared drift relative to Titjikala that Tiwi, Galiwin'ku and Yarrabah (here represented by Tiwi only) have for PNG is due to admixture or population structure with PNG such that some regions share greater ancestry with the Australian samples than others, then some points will deviate from this trend line, while others will not. No PNG samples appear to deviate from this trend line, suggesting the shared ancestry of PNG groups with Tiwi and Titjikala (and presumably Galiwin'ku and Yarrabah) is uniform across PNG.

Supplementary Note 5: Demographic modelling of the historical relationships within Australia

Taking advantage of recent developments in the rapid simulation and storage of genetic data[33] and approximate Bayesian computation via Random Forests (ABC-RF)[34,35], we developed an approach that both compares plausible demographic scenarios and allows inference of model parameters. Specifically, we compare a range of plausible phylogenetic trees that relate the four Australian and a single Highland Papuan population to each other based on genetic data from samples from those populations. Based on the most likely scenario, we estimate the parameters of divergence times, effective population sizes, bottlenecks and migration rates between populations.

Plausible demographic scenarios

While there are 105 possible tree topologies that relate these five groups to each other, our other analyses offer several constraints. We note that constraining the problem was necessary for computational reasons. The Relative Cross-coalescent analysis indicates that the five highland Papuan communities behaved as a single population as recently as 10 kya and that in general the separation between Australian and Papuan populations predates separation within Australia. These observations support the inclusion of a single Papuan sample as the outgroup.

The hierarchical clustering and UMAP analyses presented in Fig. 2 indicate substantial shared ancestry between Yarrabah and Titjikala, and while migration may contribute to these patterns, these observations are inconsistent with either Yarrabah forming the outgroup to the other three Australian groups, or Yarrabah forming a clade with the Tiwi population.

The F_3 and F_4 analyses presented in Fig. 3 and Supplementary Note 4 are also inconsistent with Galiwin'ku forming an outgroup to the other three Australian groups.

This constrains our analysis to seven plausible population topologies (Fig. 4A) that can be grouped according to common features; topologies 1 and 2 place the geographically proximate Island populations of Galiwin'ku and Tiwi in a subclade; while topologies 4, 5 and 6 group the four Australian populations according to language family, i.e., Tiwi forms the Australian outgroup.

Analysed genomic regions and recombination map

Our approach compares a set of summary statistics observed in real data with those obtained from the simulations that match in sample size and genomic coverage. We restrict our sample to 70 haplotypes from 35 individuals that include all populations and maximises the common genomic intersection after ancestry masking. This sample includes 5 individuals from Titjikala, 5 from Yarrabah, 10 from Galiwin'ku, 10 from Tiwi and 5 individuals from the highland Bundi region of PNG.

We restrict our analysis to 17 large genomic tracts from chromosomes 1 to 6 with a total length of 291 Mb that were inferred to be of Indigenous ancestry across all 70 haplotypes. To allow efficient simulation with msprime[36], genomic regions were concatenated and the recombination map[37] adjusted to include values of 0.5 at inter-segment boundaries.

Summary Statistics

Given that ABC-RF is robust to the inclusion of noisy and uninformative summary statistics[34], we included both the raw values and second and third moments of each F_3 and F_4 statistic, and the measures of within-population diversity: Tajima's D, nucleotide diversity and counts of segregating sites. Each statistic was calculated for all possible combinations of populations over 1-Mb windows.

This choice of statistics was in part motivated by the possibility they would allow inferences of migration rates, which turned out not be the case.

Statistics were computed directly on the tree sequence tables using the tskit package[38] for simulations and using ADMIXTOOLS[24] for the F statistics and plink[23] for the within-population statistics on the NCIG + PNG dataset. Concordance between approaches was confirmed. We note however, that we were unable to produce consistent F_2 values with tskit and ADMIXTOOLS, and thus did not include these statistics.

Prior Distributions on Parameters

In general, wide priors were set on most distributions, especially those pertaining to events happening further back in time, except for migration rates, which were set to be low in line with prior knowledge about the extreme isolation of these chosen groups observed in our population structure analyses. Migration rates between population pairs were specified for the entire periods over which the populations existed and allowed for the possibility of no migration.

Demographic parameters that are common to multiple topologies were given the same distributions for each topology (or as close as was possible). Priors on modern-day population sizes were informed by our IBDNe analyses and priors on ancient population sizes were chosen to be diffuse. The islandisation of the Galiwin'ku and Tiwi was modelled to allow a bottleneck occurring 6-12 kya, informed by our IBDNe analyses, with population splits constrained to occur prior.

All scenarios model the split between Papuan and Australian populations as occurring up to 65 kya, as supported by the mitochondrial analysis here and reported previously[39].

Priors and posterior distributions for key parameters are shown in *Supplementary Figures 2 – 4*. Full details of the prior distributions used are available on request.

Several lines of evidence suggested the possibility of recent admixture from a Papuan, Papuan related or Melanesian population into the Yarrabah population. This included the ADMIXTURE analysis at K=7 (*Extended Data Figure 3)*, the MDS analysis (*Extended Data Figure 2C)*, and previously published Y-

Chromosomal analysis[40] (also supported by our unpublished work) and genome wide analysis[3]. This additional migration parameter was modelled as a single discrete pulse of migrants arriving 3-7 generations ago. This additional parameter does not preclude the inference of migration at any other time point between all population pairs.

Model fitting and parameter estimation

Simulations were performed using a development version of msprime[36,41], using a discretised model of the genome (repeat mutations permitted), and a generation time of 29 years as per previous studies[3,5,42].

50,000 simulations were run for each of the seven scenarios, allowing a uniform prior probability across topologies. In addition to the summary statistics described above, we added coefficients from the axes of a linear discriminant analysis generated from the summary statistics.

The *abcrf* package (v1.9)[35] was used to fit an ABC-RF model to our training set of 350,000 simulations, based on a random forest of 1,000 trees. The trained model was then used to infer the most probable scenario from the summary statistics taken from the NCIG + PNG dataset. We note that while ABC-RF does not assign a posterior probability to each topology (only to the most probable), the advantages of ABC-RF outweigh this limitation, in our view.

Parameter inference was carried out on the most likely topology (number 4 in Fig. 4).

We further investigated the alternate scenarios that populations group by geographic distance (the Island populations of Galiwin'ku and Tiwi form a subclade) or language family (Tiwi forms an outgroup) by training two additional ABC-RF models after reclassifying simulations into two categories. The model testing geography grouped topologies 1 and 2 verses the rest. The model testing language grouped topologies 4, 5 and 6 verses the rest. In both cases an equal number of simulations were assigned to each class to allow an equal prior probability of 0.5.

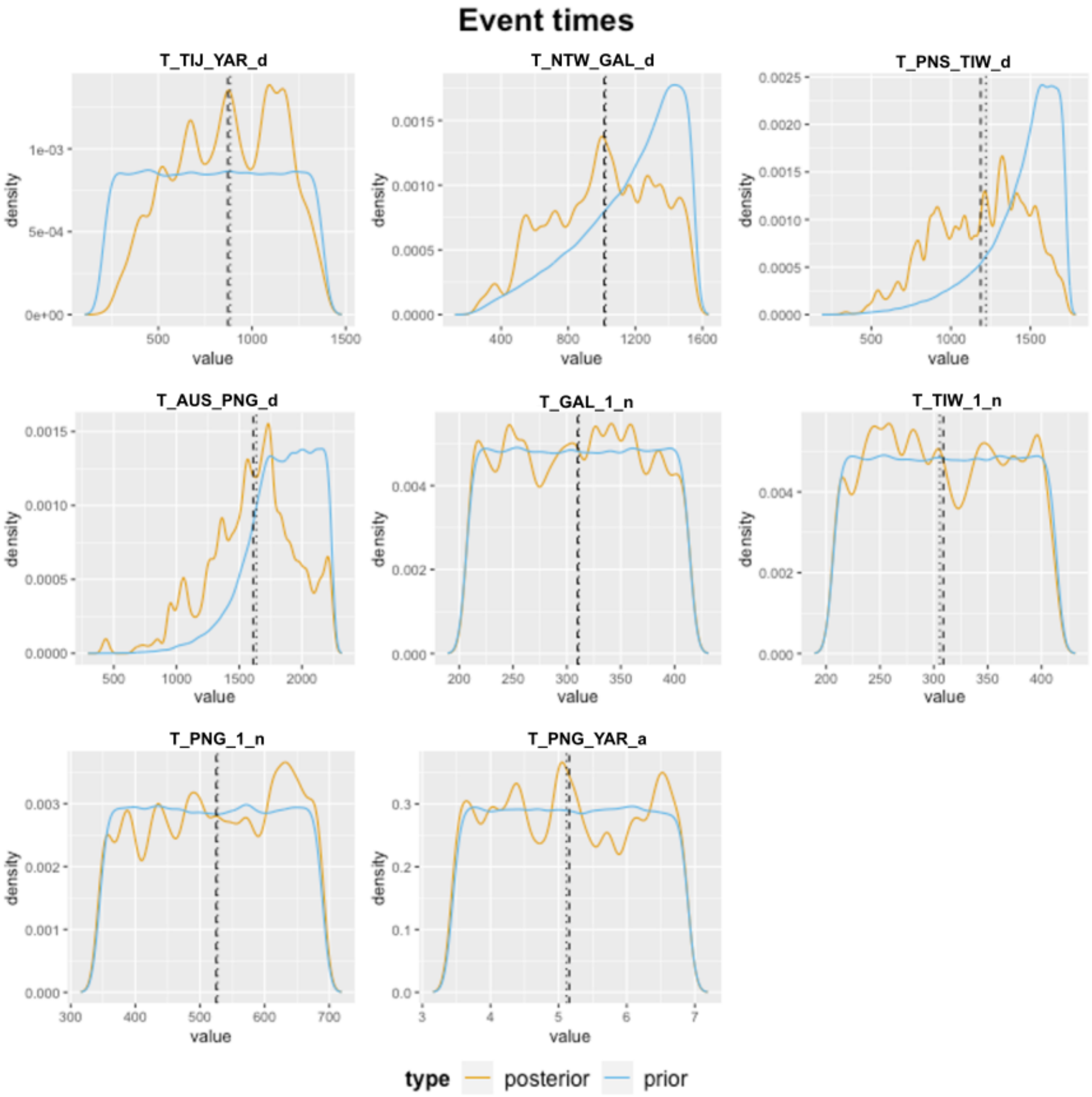

Supplementary Figure 2. ABC analysis population divergence, bottleneck and admixture time estimates. Prior (blue) and posterior (orange) densities of event times in Scenario 4 (Fig. 4). X-axis is generations ago. Posterior expectations are black dashed lines, while posterior medians are dotted lines. The panels are for each event with labels: T_TIJ_YAR_d = Titjikala – Yarrabah split time; T_NTE_GAL_d = Non Top-End (Ancestral to Titjikala and Yarrabah) – Galiwin'ku split time; T_PNS_TIW_d = Pama-Nyungan Speakers (Ancestral to Galiwin'ku, Titjikala and Yarrabah) – Tiwi split time; T_AUS_PNG_d = Australian (Ancestral to all Australian samples) – PNG split time; T_GAL_1_n = Galiwin'ku bottleneck time; T_TIW_1_n = Tiwi bottleneck time; T_PNG_1_n = PNG bottleneck time; T_PNG_YAR_a = time of recent admixture from PNG into Yarrabah.

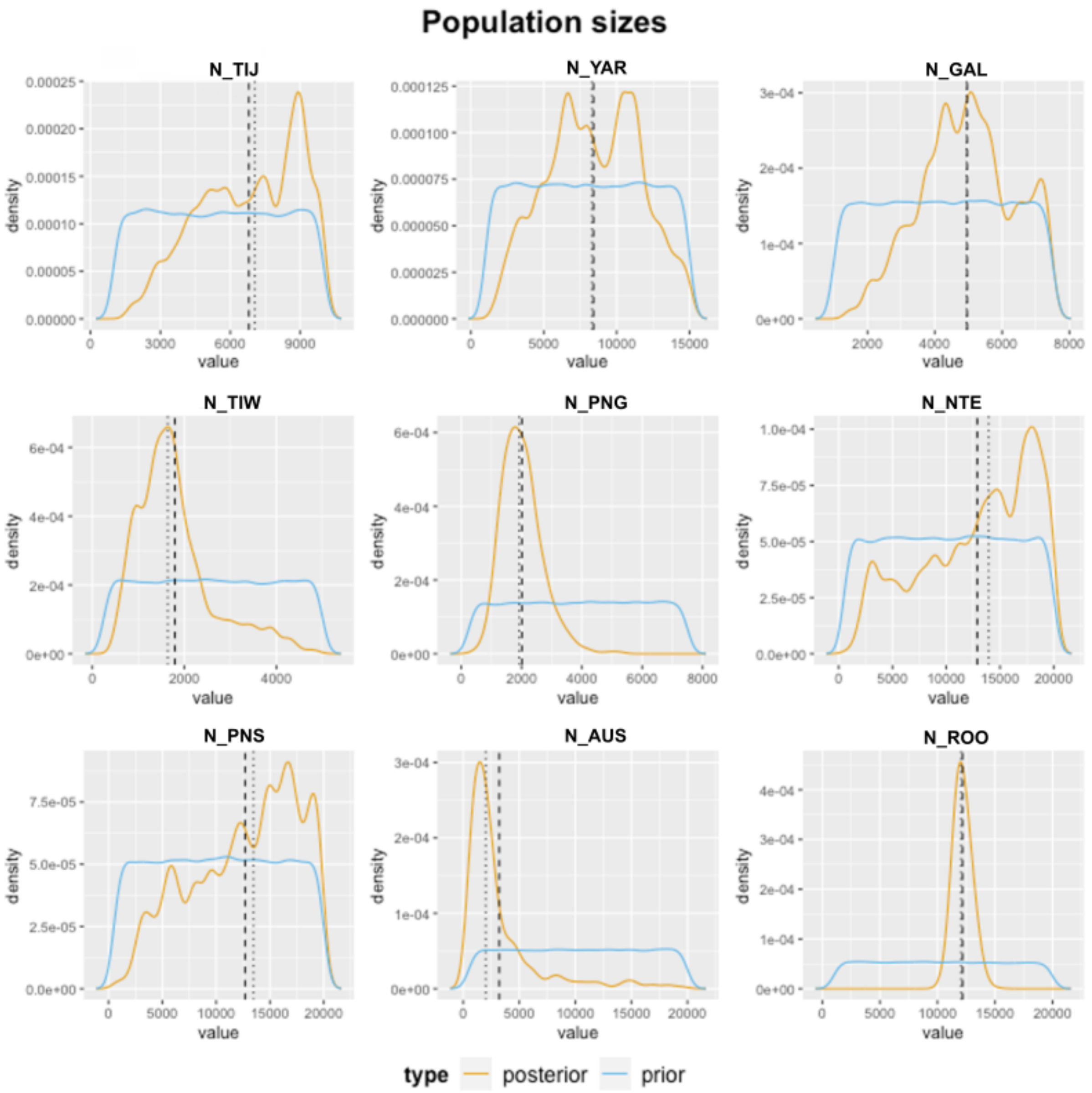

Supplementary Figure 3. ABC analysis effective population size estimates for Scenario 4 (Fig. 4A). Prior (blue) and posterior (orange) densities for the current and ancestral populations shown in Fig. 4B. X-axis is N_e. Posterior expectations are shown with black dashed lines, while posterior medians are shown with black dotted lines. The panels are for each populations with labels: N_TIJ = Titjikala; N_YAR = Yarrabah, N_GAL = Galiwin'ku; N_TIW = Tiwi; N_PNG = PNG; N_NTE = Non Top-End (Ancestral to Titjikala and Yarrabah); N_PNS = Pama-Nyungan Speakers (Ancestral to Galiwin'ku, Titjikala and Yarrabah); N_AUS = Australian (Ancestral to all Australian samples); N_ROO = Root population (Ancestral to Australia and PNG).

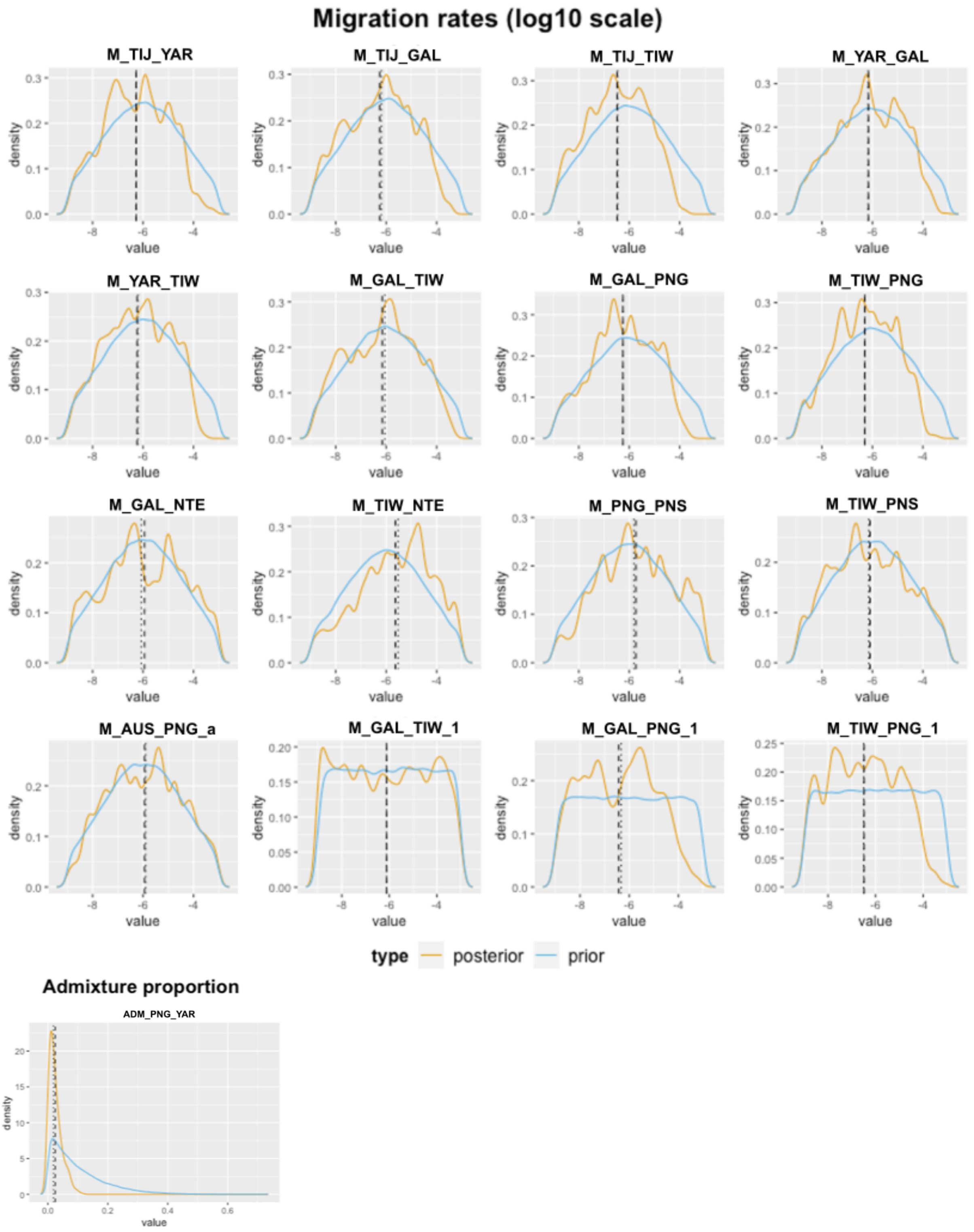

Supplementary Figure 4. ABC analysis of migration rate estimates (x-axis, $\log_{10}$ scale). Prior (blue) and posterior (orange) densities of event times in Scenario 4 (Fig. 4). Posterior expectations are shown with black dashed lines, while posterior medians are shown with dotted lines. There are panels for migration rates between pairs of populations, with populations labelled: TIJ = Titjikala; YAR = Yarrabah, GAL = Galiwin'ku; TIW = Tiwi; PNG = PNG; NTE = Non-Top-End (Ancestral to Titjikala and Yarrabah); PNS = Pama-Nyungan Speakers (Ancestral to Galiwin'ku, Titjikala and Yarrabah); AUS = Australian (Ancestral to all Australian samples); The three parameters labelled with "_1" denote migration prior to the modelled bottlenecks in these populations.

Those labelled with "_a" refer to the ancestral populations. (M = migration; ADM = admixture). The admixture proportion plot is for recent admixture from PNG into Yarrabah (3-7 generations ago).

Supplementary Table 2. Estimated event times for Scenario 4 in generations (29 years / generation assumed for conversion to years).

Parameter	Mean	Median	HPDI 95
$T^D_{TIJ,YAR}$	870.483	883.932	(353.088, 1323.996)
$T^D_{NTE,GAL}$	1013.669	1023.783	(384.183, 1510.133)
$T^D_{PNS,TIW}$	1187.140	1220.157	(582.490, 1654.833)
$T^D_{AUS,PNG}$	1612.113	1636.141	(934.397, 2212.412)
T^1_{GAL}	309.873	311.127	(212.465, 409.066)
T^1_{TIW}	308.819	305.513	(212.359, 407.419)
T^1_{PNG}	524.883	527.205	(352.581, 683.682)
$T^A_{PNG,YAR}$	5.155	5.116	(3.530, 6.810)

T^D = Time of split, T^1 = Time of bottleneck, *TIJ* = Titjikala, *YAR* = Yarrabah, *NTE* = Non-Top-End (Ancestral to *TIJ* and *YAR*), *GAL* = Galiwin'ku, *PNS* = Pama-Nyungan speakers (Ancestral to *TIJ*, *YAR* and *GAL*), T^A = Time of Admixture from *PNG* into Yarrabah, HPDI = Highest posterior density interval.

Supplementary Table 3. Estimated effective population sizes for Scenario 4.

Parameter	Mean	Median	HPDI 95
N_{TIJ}	6571.002	6618.472	(2503.0240, 9823.022)
N_{YAR}	9746.144	9698.641	(3787.4253, 14811.338)
N_{GAL}	5136.901	5184.649	(2357.7013, 7394.401)
N_{TIW}	1611.770	1401.426	(693.8842, 3776.523)
N_{PNG}	1967.810	1865.258	(994.6391, 3478.643)
N_{NTE}	12565.143	13234.093	(2379.9880, 19655.238)
N_{PNS}	11776.713	12118.454	(2666.8390, 19654.034)
N_{AUS}	3421.676	2010.928	(549.7647, 16303.407)

AUS = Ancestral to the four Australian populations. All labels *as per Supplementary Figure 3*.

Demographic inference with AdmixtureBayes

As an alternate means of inferring demographic parameters, we implemented the recently published AdmixtureBayes algorithm (version 0.3)[43]. This approach uses MCMC to estimate likely population topologies, branch lengths and admixture proportions based on comparisons of observed and expected allele frequency covariance values between populations. To ensure an unbiased comparison of the two methods, we ran AdmixtureBayes using the same subset of samples and genomic regions as were used for the ABC inference (details above). This dataset included 5 samples from each of Yarrabah, Titjikala and PNG (HL), and ten from Galiwin'ku and Tiwi. PNG (HL) was set as the outgroup population as required by AdmixtureBayes. This dataset was filtered on the basis of minor allele frequency and linkage disequilibrium, yielding 150,861 biallelic SNPs for subsequent analysis.

AdmixtureBayes was run with default settings, aside from the 'n' parameter, which controls the number of MCMC iterations, which we increased to 100,000. Convergence was assessed based on the output of the

‘EvaluateConvergence.R’ script supplied with the AdmixtureBayes package. The trees sampled during the MCMC procedure were downsampled using default values of the ‘burn_in_fraction’ and ‘thinning_rate’ parameters, and we explored the top scoring trees (along with their branch lengths) and consensus trees.

The best supported topology (Tiwi (Galiwin’ku (Titjikala, Yarrabah))), with a posterior probability of 18.6% (*Supplementary Figure 5A*), was also the most supported topology under the ABC analysis (topology number 4 in Fig. 4). The next most supported topologies received posterior probabilities of 13.9 and 10.9% (*Supplementary Figure 5A*).

Consistent with the results of the ABC analysis, the most likely initial division of Australian populations separates Tiwi from the three remaining groups, as 37.6% of trees sampled have the (Galiwin’ku, Yarrabah, Titjikala) clade. Also consistent with the ABC analysis was the grouping of Titjikala and Yarrabah, which occurred in 32.7% of the sampled trees. Overall, however, the data did not provide strong support for any grouping of Indigenous Australian populations, and consensus trees generated at 50, 75, 90 and 95% node support thresholds all yielded ‘star’ topologies, in which all populations descend from one ancestral Australian group (*Supplementary Figure 5B*).

Interestingly, admixture events were rare in the best supported topologies, with none inferred amongst the 15 top ranking trees. Estimating branch lengths from the best supported scenarios revealed short internal branches and long terminal branches (*Supplementary Figure 5A*). These internal branches were consistently an order of magnitude or more lower than the length of the terminal branches, and show that the extent of genetic drift shared between Indigenous populations is minimal compared to the drift they experienced since divergence from one another. These features support inferences made in other analyses which emphasise the strong degree of genetic drift which these Indigenous Australian groups have undergone, and the limited role of migration between communities inferred using ABC.

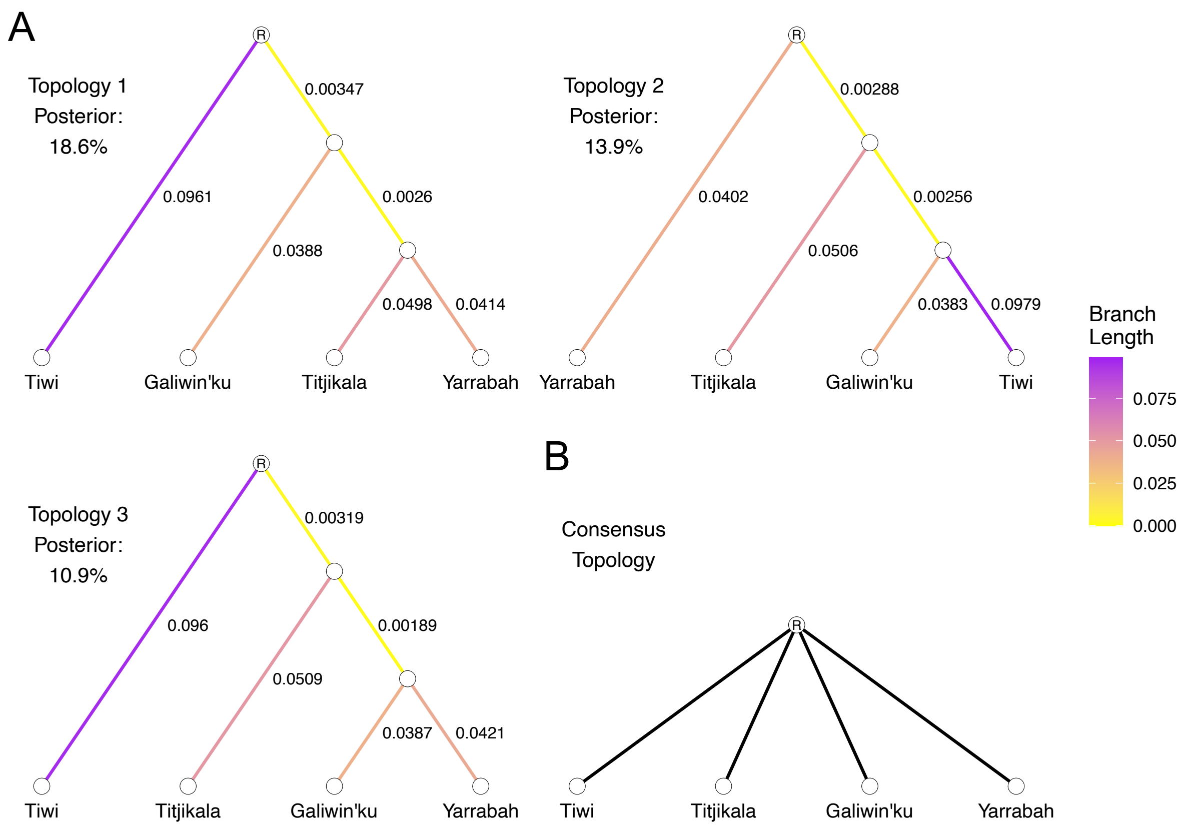

Supplementary Figure 5. **A.** The three best supported topologies when applying AdmixtureBayes to the same subsample of individuals and genomic regions as used for the ABC analysis (Main Text, *Figure 4*). Branch colours are scaled according to the amount of genetic drift which occurred on that branch, with values printed next to each branch. **B.** Consensus tree generated using a posterior node support value of 90%.

Supplementary Note 6: Historic Autosomal Effective Population Size and Isolation

We used IBDNe[44] to infer recent effective population sizes (N_e), MSMC2[45] to infer N_e over deeper time periods, and MSMC2 to infer genetic isolation between population pairs.

Recent Effective Population Sizes with IBDNe

IBD segments were inferred for all NCIG + PNG individuals (without ancestry masking or relatedness filtering) using RefinedIBD[46] after filtering to exclude variants with a count of strictly less than 8. Short gaps and breaks in IBD segments (potentially caused by phasing or sequencing errors) were removed with the ‘merge-ibd-segments.17Jan20.102.jar’ utility available with the RefinedIBD software.

For the Tiwi, Galiwin’ku, Titjikala and PNG (HL) related individuals (2nd degree and closer) and those with greater than 5% non-Indigenous ancestry were removed (as was done elsewhere in this study), and IBDNe was run on unrelated samples with default parameters, except for a ‘mincm’ value of 1.5cM instead of 2cM, as the RefinedIBD software is capable of accurately identifying segments this small from WGS data[44,46]. The HapMap GRCh38 specific recombination map[31] obtained with the RefinedIBD software was used. 95% confidence intervals were obtained by bootstrapping (automatically calculated by the software).

Ancestry specific N_e was estimated[47] for all unrelated individuals from the admixed populations of Yarrabah and PNG(Is.) using RefinedIBD tracts (filtered as above) and local ancestry inferred from RFMIX. We only report N_e for the Indigenous component of each population (Fig. 5A and *Supplementary Figure 6*).

The above filtering results in the following sample sizes; Tiwi, 34; Galiwin'ku, 13; Titjikala, 6; Yarrabah, 31; PNG (HL), 25; PNG (Is.), 28. A recent study demonstrated a sample size of 30 was sufficient for accurate N_e estimation with IBDNe[19]. We note that our sample sizes for Galiwin'ku and Titjikala are small, which may affect inference in these populations with this method.

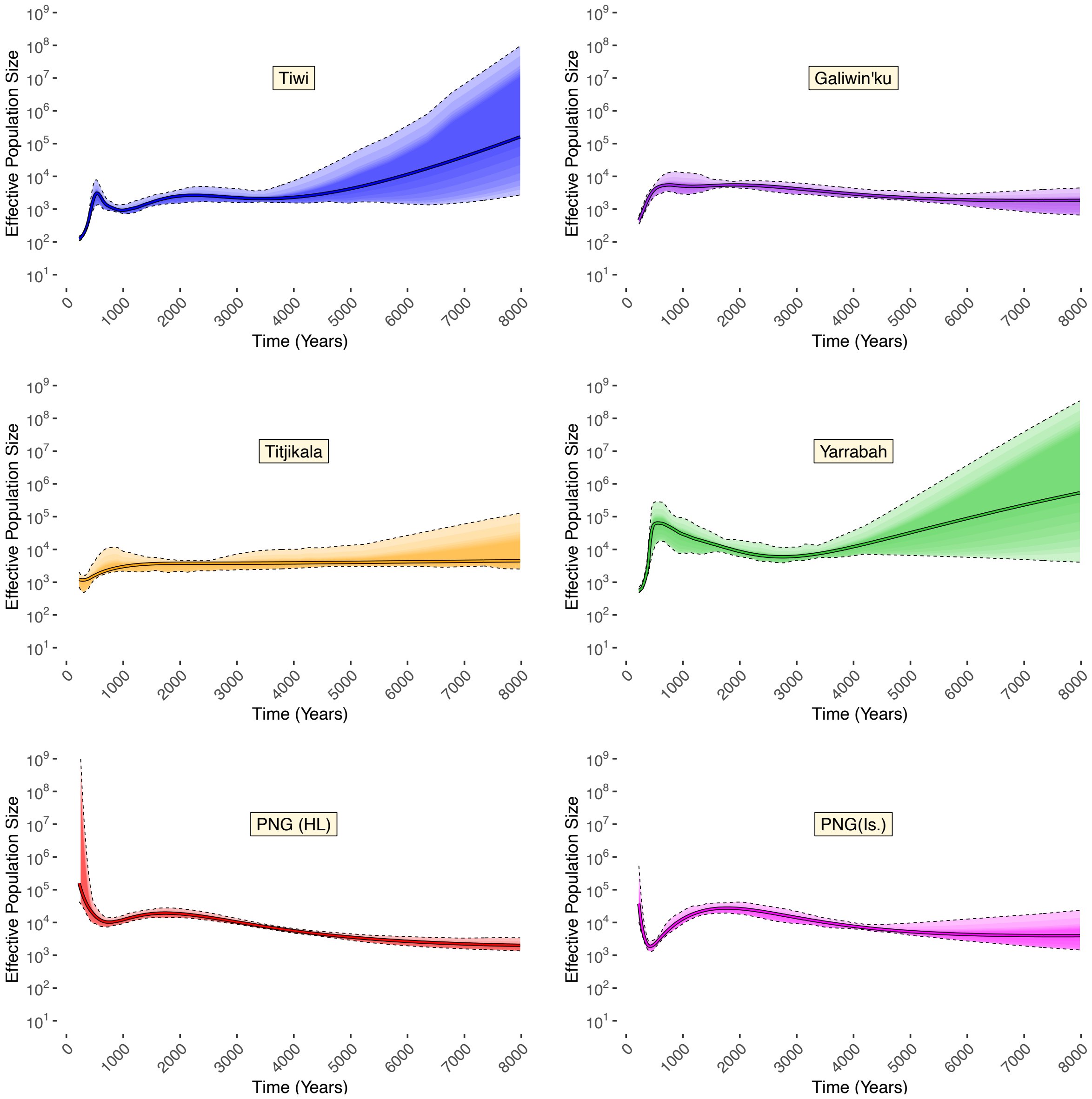

Supplementary Figure 6. Mean effective population size estimated for the last 6000 years (assuming generation time of 28 years) for all NCIG + PNG populations inferred using IBDNe. Shaded regions show bootstrap confidence intervals. Note that Yarrabah and PNG (Is.) population size estimates were inferred after applying the 'ancestry specific effective population size' pipeline described by Browning et al. (2018). Only results for the 'Indigenous' components (meaning Australian and Papuan respectively) for these populations are shown.

Long Term Effective Population Sizes MSMC2

MSMC2 (v.2.1.2)[45] was used to infer within population N_e estimates after filtering individuals for relatedness (as above) and removing individuals with any evidence of non-Indigenous ancestry (a more stringent criteria than used elsewhere). The eight Tiwi individuals with evidence of non-Tiwi Indigenous ancestry were excluded

and only the 10 individuals with the least non-Indigenous ancestry from Yarrabah were retained. For these Yarrabah samples we also applied the RFMIX ancestry tracts coordinates to mask regions where either or both haplotypes are inferred to be non-Indigenous.

The algorithm was run as recommended[45] with exceptions discussed below. Inference was run across all autosomes using 8 phased haplotypes from 4 randomly sampled individuals from each population. This was repeated five times with a unique set of individuals from each population (although some individuals may appear in more than one sampled set) and the mean N_e across replicates was plotted against the mean of the mid-point time intervals (Fig. 5B). Time boundaries were converted to years with a mutation rate of 1.25e-8 and a generation time of 29 years as per previous studies[3,5,42].

We used bamCaller.py from MSMC tools (default parameters; coverage within 0.5x to 2x the per-sample average, mapQ >20 and baseQ > 20), for each sample to generate a mask of low coverage genomic regions. Per-sample average read-depth was obtained from CRAM files using samtools depth. These masks remove on average 14.8% of the genome (range 13.5% to 19.7%). We note our samples have an average read-depth greater than 30x, more than the 20x reported as sufficient for MSMC2[45].

We generated the common mapability mask for each chromosome of GRCh38 based on a kmer length of 100bp, setting a conservative threshold given our paired-end reads are 150-151 bp. This mask was generated as recommended(“http://lh3lh3.users.sourceforge.net/snpable.shtml,” n.d.) with stringency of 0.5 (default setting). The resulting FASTA files were converted to BED format with makeMappabilityMask.py from MSMC-tools[49]. This mask removed 686 Mb, or 21.4%, of the reference genome.

There is a strong intersection between the per-sample low coverage masks and the common mapability mask, with the intersection of available common regions for all sets of 8 haplotypes ranging from 73.0% and 77.6% of the genome (3.4 to 4.4 million segregating sites available or analysis).

While the ancestry masks remove between 22.7% and 57.0% of each Yarrabah genome, the intersection of all masks for each set of 8 Yarrabah haplotypes resulted in the available sites dropping to between 3.0% and 29.0% of the genome. We therefore repeated the above analysis using 10 replicates of 4 haplotypes per population. This resulted in the retention of between 18% and 73% of the genome for each Yarrabah set but yielded similar N_e estimates over all time periods except the last 10,000 years for Yarrabah and 1,000 years for the other populations (data not shown).

Single sample VCFs were extracted from our multi-sample phased VCF generated using the phase informative read extension of SHAPEIT2 (v2.12, discussed in methods). This has the advantage of retaining and phasing all variable sites within an individual, not just those present in a reference panel. As discussed in Supplementary Note 2, using our Chromium 10X data we estimate on average one switch error every 91 kb in Titjikala, 293 kb in Yarrabah, 322 kb in Galiwin’ku and 1,870 kb in Tiwi. These error rates are less than previously reported for two Indigenous Australian samples (one error every 34 kb and 83 kb; Mallick et al. 2016) and below the levels reported to adversely affect rCCR estimates in simulations[3,5].

Population Isolation with MSMC2 relative cross coalescent rate

To estimate separation times, we used MSMC2 to calculate the relative cross coalescence rate (rCCR) for all 45 pairs of populations. For each pair, we ran 10 replicates of 4 phased haplotypes per population (two individuals). Parameters and masks were applied as described for N_e estimation above.

The midpoint rCCR was estimated for each replicate using linear interpolation. Differences in midpoint rCCR were tested via one-way ANOVA in R, with pairwise comparisons made using Tukey Honest Significant Differences in R (TukeyHSD). All comparisons that were significant for this test remained significant using a pairwise t-test, correcting for multiple testing using the Benjamini-Hochberg method.

We note that MSMC2 is sensitive to switch errors in phased haplotypes, resulting in an inflation of split times. In our case, the oldest split times we observe between population pairs involve Tiwi, a population for which we estimate our lowest switch error rate, almost 10 times less than the rate shown to bias split time in simulations (Mallick et al. 2016 - Figure S9.2)[5]. Thus, while other factors may bias these results, we believe switch error does not.

Supplementary Note 7: Mitochondrial Genetic Structure and Diversity

Mitochondrial variants were called separately to autosomal variants to account for the haploid nature of the molecule and to carefully validate the variant call pipeline for false positives. Variants were called using GATK 'HaplotypeCaller' applying a mapping quality cut-off of 30 and a base quality cut-off of 20[50]. Joint genotyping was not used, as it led to a reduction in the number of rare alternate genotype calls, particularly in underrepresented haplotypes. The variant call pipeline was validated through analysis of filtered allele depth, and the number of alternate alleles called per mitogenome when comparing individuals from the three WGS cohorts used from the NCIG + PNG dataset. Very similar distributions of allele depth and counts of alternate sites within the same haplogroup show no apparent batch effect. As a final means of validation, genotype concordance was assessed in maternal parent-offspring pairs. Of the three mother-offspring relationships analysed, no mismatching genotype calls were identified, indicating the variant call pipeline was robust.

Mitochondrial phylogenies were inferred using BEAST[51], allowing a relaxed uncorrelated lognormal clock, a Coalescent Bayesian Skyline tree prior, and the mitochondrial mutation rate of Fu et al. (2014)[52], parameters closely matching those used in a prior study of human mitochondrial data[39]. BEAST was run using 30 million MCMC iterations, sampling every 1000th tree. Mixing of model parameters was analysed using TRACER (v1.7)[51] by inspecting effective sample size (ESS) values for model parameters and judging a run to be well mixed when ESS values exceeded 200. A maximum clade credibility tree (MCC) was produced using TreeAnnotator from the posterior distribution of trees sampled in the BEAST run. Tree visualisation was carried out using ggtree[53], with comparable results obtained with PhyloTree[54]. Mitochondrial lineages were assigned using HaploGrep2[55].

Previously published Australian and Melanesian mitochondrial sequences were included in the phylogeny to illustrate the most recent points of coalescence between Australian and PNG lineages[39,56–63]. To explore the frequency of the three mitochondrial haplogroups with a recent coalescence time to Melanesian lineages (N13, Q2 and P3) across the Australian continent, data from previous studies of Indigenous mitochondrial variation were collated[3,57,63–66]. This dataset consisted of the frequencies of key Australian and Melanesian lineages (O, S, N13, P3, P4, P5, P6, P7, P8, R12, M41, Q, E1a2), and excluded any of European or East Asian origin.

Statistical tests of association were performed to explore the relationship the distribution of these recently coalescing haplogroups may have to populations with elevated shared drift with PNG as inferred from autosomal data. Australian populations used for the combined analysis were classified by both language group and geographic region. The language group classification divided groups on whether they spoke a non-Pama-Nyungan or Pama-Nyungan language, or whether their language family was 'unknown'. The geographical classification divided groups into Top End/Kimberley, and non-Top End (encompassing all remaining populations). This division was chosen to roughly describe the pattern of shared drift with PNG observed in the northern populations of Australia in the F-statistic analysis, and the lack of an apparent shared drift with PNG of any of the more southerly populations described previously[3]. Mann Whitney U tests were used to test for differences in the frequency of each of the three recently coalescing lineages when considering these two criteria for classification. These tests were performed using the 'stats' package in R.

Supplementary Information References

1. Australian National University. *National Centre for Indigenous Genomics Statute. https://www.legislation.gov.au/Details/F2021L00183* (2021).
2. Thomson, R. J. *et al.* New genetic loci associated with chronic kidney disease in an indigenous Australian population. *Front Genet* **10**, (2019).
3. Malaspinas, AS. *et al.* A genomic history of Aboriginal Australia. *Nature* **538**, 207–214 (2016).
4. Vernot, B. *et al.* Excavating Neandertal and Denisovan DNA from the genomes of Melanesian individuals. *Science* **352**, 235–239 (2016).
5. Mallick, S. *et al.* The Simons Genome Diversity Project: 300 genomes from 142 diverse populations. *Nature* **538**, 201–206 (2016).
6. Auton, A. *et al.* A global reference for human genetic variation. *Nature* **526**, 68–74 (2015).
7. Byrska-Bishop, M. *et al.* High-coverage whole-genome sequencing of the expanded 1000 Genomes Project cohort including 602 trios. *Cell* **185**, 3426-3440.e19 (2022).
8. Bergström, A. *et al.* A Neolithic expansion, but strong genetic structure, in the independent history of New Guinea. *Science* **357**, 1160–1163 (2017).
9. Li, H. Aligning sequence reads, clone sequences and assembly contigs with BWA-MEM. (2013).
10. Danecek, P. *et al.* Twelve years of SAMtools and BCFtools. *Gigascience* **10**, (2021).
11. Tischler, G. & Leonard, S. biobambam: tools for read pair collation based algorithms on BAM files. *Source Code Biol Med* **9**, 13 (2014).
12. Poplin, R. *et al.* Scaling accurate genetic variant discovery to tens of thousands of samples. doi:10.1101/201178.
13. Delaneau, O., Howie, B., Cox, A. J., Zagury, J. F. & Marchini, J. Haplotype estimation using sequencing reads. *Am J Hum Genet* **93**, 687–696 (2013).
14. Alexander, D. H., Novembre, J. & Lange, K. Fast model-based estimation of ancestry in unrelated individuals. *Genome Res* **19**, 1655–1664 (2009).
15. Maples, B. K., Gravel, S., Kenny, E. E. & Bustamante, C. D. RFMix: A discriminative modeling approach for rapid and robust local-ancestry inference. *Am J Hum Genet* **93**, 278–288 (2013).
16. https://github.com/armartin/ancestry_pipeline.
17. Pierron, D. *et al.* Strong selection during the last millennium for African ancestry in the admixed population of Madagascar. *Nat Commun* **9**, 932 (2018).
18. Conley, A. B. *et al.* A Comparative Analysis of Genetic Ancestry and Admixture in the Colombian Populations of Chocó and Medellín. *G3 Genes|Genomes|Genetics* **7**, 3435–3447 (2017).
19. Mooney, J. A. *et al.* Understanding the Hidden Complexity of Latin American Population Isolates. *Am J Hum Genet.***103**, 707–726 (2018).
20. Xue, A. *et al.* Genome-wide association analyses identify 143 risk variants and putative regulatory mechanisms for type 2 diabetes. *Nat Commun* **9**, (2018).
21. Lawson, D. J., Hellenthal, G., Myers, S. & Falush, D. Inference of Population Structure using Dense Haplotype Data. *PLoS Genet* **8**, e1002453 (2012).
22. Patterson, N., Price, A. L. & Reich, D. Population structure and eigenanalysis. *PLoS Genet* **2**, 2074–2093 (2006).
23. Purcell, S. *et al.* PLINK: A Tool Set for Whole-Genome Association and Population-Based Linkage Analyses. *Am J Hum Genet.***81**, 559–575 (2007).
24. Patterson, N. *et al.* Ancient admixture in human history. *Genetics* **192**, 1065–1093 (2012).
25. Browning, S. R. *et al.* Local Ancestry Inference in a Large US-Based Hispanic/Latino Study: Hispanic Community Health Study/Study of Latinos (HCHS/SOL). *G3 Genes|Genomes|Genetics* **6**, 1525–1534 (2016).
26. R Core Team. R: A language and environment for statistical computing. *R Foundation for Statistical Computing, Vienna, Austria. URL https://www.R-project.org/* (2021).
27. Browning, B. L. & Browning, S. R. Improving the Accuracy and Efficiency of Identity-by-Descent Detection in Population Data. *Genetics* **194**, 459–471 (2013).
28. Browning, S. R. & Browning, B. L. Identity by Descent Between Distant Relatives: Detection and Applications. *Annu Rev Genet* **46**, 617–633 (2012).
29. McInnes, L., Healy, J., Saul, N. & Großberger, L. UMAP: Uniform Manifold Approximation and Projection. *J Open Source Softw* **3**, 861 (2018).
30. Leslie, S. *et al.* The fine-scale genetic structure of the British population. *Nature* **519**, 309–314 (2015).

31. International HapMap 3 Consortium. Integrating common and rare genetic variation in diverse human populations. *Nature* **467**, 52–58 (2010).
32. Peter, B. M. Admixture, population structure, and f-statistics. *Genetics* **202**, 1485–1501 (2016).
33. Kelleher, J. *et al.* Inferring whole-genome histories in large population datasets. *Nat Genet* **51**, 1330–1338 (2019).
34. Raynal, L. *et al.* ABC random forests for Bayesian parameter inference. *Bioinformatics* **35**, 1720–1728 (2019).
35. Pudlo, P. *et al.* Reliable ABC model choice via random forests. *Bioinformatics* **32**, 859–866 (2016).
36. Kelleher, J., Etheridge, A. M. & McVean, G. Efficient Coalescent Simulation and Genealogical Analysis for Large Sample Sizes. *PLoS Comput Biol* **12**, e1004842 (2016).
37. A second generation human haplotype map of over 3.1 million SNPs. *Nature* **449**, 851–861 (2007).
38. Ralph, P., Thornton, K. & Kelleher, J. Efficiently Summarizing Relationships in Large Samples: A General Duality Between Statistics of Genealogies and Genomes. *Genetics* **215**, 779–797 (2020).
39. Tobler, R. *et al.* Aboriginal mitogenomes reveal 50,000 years of regionalism in Australia. *Nature* **544**, 180–184 (2017).
40. Bergström, A. *et al.* Deep Roots for Aboriginal Australian Y Chromosomes. *Current Biology* **26**, 809–813 (2016).
41. Baumdicker, F. *et al.* Efficient ancestry and mutation simulation with msprime 1.0. *Genetics* **220**, (2022).
42. Bergström, A. *et al.* Insights into human genetic variation and population history from 929 diverse genomes. *Science* **367**, (2020).
43. Nielsen, S. V. *et al.* Bayesian inference of admixture graphs on Native American and Arctic populations. *PLoS Genet* **19**, e1010410 (2023).
44. Browning, S. R. & Browning, B. L. Accurate Non-parametric Estimation of Recent Effective Population Size from Segments of Identity by Descent. *Am J Hum Genet.***97**, 404–418 (2015).
45. Schiffels, S. & Wang, K. MSMC and MSMC2: The Multiple Sequentially Markovian Coalescent. in 147–166 (2020). doi:10.1007/978-1-0716-0199-0_7.
46. Browning, B. L. & Browning, S. R. Detecting Identity by Descent and Estimating Genotype Error Rates in Sequence Data. *Am J Hum Genet.***93**, 840–851 (2013).
47. Browning, S. R. *et al.* Ancestry-specific recent effective population size in the Americas. *PLoS Genet* **14**, e1007385 (2018).
48. http://lh3lh3.users.sourceforge.net/snpable.shtml.
49. https://github.com/stschiff/msmc-tools/blob/master/README.md.
50. McKenna, A. *et al.* The Genome Analysis Toolkit: A MapReduce framework for analyzing next-generation DNA sequencing data. *Genome Res* **20**, 1297–1303 (2010).
51. Drummond, A. J. & Rambaut, A. BEAST: Bayesian evolutionary analysis by sampling trees. *BMC Evol Biol* **7**, 214 (2007).
52. Fu, Q. *et al.* Genome sequence of a 45,000-year-old modern human from western Siberia. *Nature* **514**, 445–449 (2014).
53. Yu, G., Smith, D. K., Zhu, H., Guan, Y. & Lam, T. T. ggtree: an r package for visualization and annotation of phylogenetic trees with their covariates and other associated data. *Methods Ecol Evol* **8**, 28–36 (2017).
54. van Oven, M. & Kayser, M. Updated comprehensive phylogenetic tree of global human mitochondrial DNA variation. *Hum Mutat* **30**, E386–E394 (2009).
55. Weissensteiner, H. *et al.* HaploGrep 2: mitochondrial haplogroup classification in the era of high-throughput sequencing. *Nucleic Acids Res* **44**, W58–W63 (2016).
56. Rasmussen, M. *et al.* An Aboriginal Australian Genome Reveals Separate Human Dispersals into Asia. *Science* **334**, 94–98 (2011).
57. Hudjashov, G. *et al.* Revealing the prehistoric settlement of Australia by Y chromosome and mtDNA analysis. *Proc Natl Acad Sci USA* **104**, 8726–8730 (2007).
58. Friedlaender, J. S. *et al.* Melanesian mtDNA Complexity. *PLoS One* **2**, e248 (2007).
59. Friedlaender, J. S. *et al.* The Genetic Structure of Pacific Islanders. *PLoS Genet* **4**, e19 (2008).
60. Ingman, M. & Gyllensten, U. Mitochondrial Genome Variation and Evolutionary History of Australian and New Guinean Aborigines. *Genome Res* **13**, 1600–1606 (2003).

61. Corser, C. A., McLenachan, P. A., Pierson, M. J., Harrison, G. L. A. & Penny, D. The Q2 Mitochondrial Haplogroup in Oceania. *PLoS One* **7**, e52022 (2012).
62. Nagle, N. *et al.* Aboriginal Australian mitochondrial genome variation – an increased understanding of population antiquity and diversity. *Sci Rep* **7**, 43041 (2017).
63. Nagle, N. *et al.* Mitochondrial DNA diversity of present-day Aboriginal Australians and implications for human evolution in Oceania. *J Hum Genet* **62**, 343–353 (2017).
64. van Holst Pellekaan, S. M., Ingman, M., Roberts-Thomson, J. & Harding, R. M. Mitochondrial genomics identifies major haplogroups in Aboriginal Australians. *Am J Phys Anthropol* **131**, 282–294 (2006).
65. Redd, A. J. & Stoneking, M. Peopling of Sahul: mtDNA Variation in Aboriginal Australian and Papua New Guinean Populations. *Am J Hum Genet.***65**, 808–828 (1999).
66. Huoponen, K., Schurr, T. G., Chen, Y.-S. & Wallace, D. C. Mitochondrial DNA variation in an Aboriginal Australian population: evidence for genetic isolation and regional differentiation. *Hum Immunol* **62**, 954–969 (2001).
