# Supplementary material for: Indigenous Australian genomes show deep structure and rich novel variation
Source: Nature. 2023 Dec 13;624(7992):593–601. doi: 10.1038/s41586-023-06831-w (PMC10733150; doi:10.1038/s41586-023-06831-w)
Supplement: Supplementary file 2 — Reporting Summary [file 41586_2023_6831_MOESM2_ESM.pdf]

nature portfolio

Corresponding author(s): Stephen Leslie

Last updated by author(s): 2023.09.14

Reporting Summary

Nature Portfolio wishes to improve the reproducibility of the work that we publish. This form provides structure for consistency and transparency in reporting. For further information on Nature Portfolio policies, see our Editorial Policies and the Editorial Policy Checklist.

Statistics

For all statistical analyses, confirm that the following items are present in the figure legend, table legend, main text, or Methods section.

n/a	Confirmed	
☐	☒	The exact sample size (*n*) for each experimental group/condition, given as a discrete number and unit of measurement
☐	☒	A statement on whether measurements were taken from distinct samples or whether the same sample was measured repeatedly
☐	☒	The statistical test(s) used AND whether they are one- or two-sided<br>*Only common tests should be described solely by name; describe more complex techniques in the Methods section.*
☐	☒	A description of all covariates tested
☐	☒	A description of any assumptions or corrections, such as tests of normality and adjustment for multiple comparisons
☐	☒	A full description of the statistical parameters including central tendency (e.g. means) or other basic estimates (e.g. regression coefficient) AND variation (e.g. standard deviation) or associated estimates of uncertainty (e.g. confidence intervals)
☐	☒	For null hypothesis testing, the test statistic (e.g. *F*, *t*, *r*) with confidence intervals, effect sizes, degrees of freedom and *P* value noted<br>*Give P values as exact values whenever suitable.*
☐	☒	For Bayesian analysis, information on the choice of priors and Markov chain Monte Carlo settings
☒	☐	For hierarchical and complex designs, identification of the appropriate level for tests and full reporting of outcomes
☐	☒	Estimates of effect sizes (e.g. Cohen's *d*, Pearson's *r*), indicating how they were calculated

Our web collection on statistics for biologists contains articles on many of the points above.

Software and code

Policy information about availability of computer code

Data collection	No novel software was used for either the collection or analysis of this data. All existing algorithms used are referenced in the text.
Data analysis	MSMC2 v.2.1.2<br>bwa mem (v0.7.17)<br>samtools (v1.9)<br>bammerge (v.2.0.95)<br>GATK (v.3.8-0)<br>SHAPEIT (v2.12)<br>ADMIXTURE v1.3<br>RFMIX v1.5.4<br>Plink v1.9<br>Admixtools v5.1<br>R (v.4)<br>RefinedIBD v102<br>UMAP v0.2.7.0<br>fineSTRUCTURE v4.0.1<br>tskit (development version, since released as v1.0)<br>msprime (v1 within tskit release)<br>abcrf (v1.9)<br>EIGENSOFT v7.2.1<br>bcftools v1.11

ggplot2 v3.3.5
ggmap v3.0.0
R 'stats' package
IBDNe
BEAST v2.6.0
Tracer v1.7
AdmixtureBayes v0.3

For manuscripts utilizing custom algorithms or software that are central to the research but not yet described in published literature, software must be made available to editors and reviewers. We strongly encourage code deposition in a community repository (e.g. GitHub). See the Nature Portfolio guidelines for submitting code & software for further information.

Data

Policy information about availability of data

All manuscripts must include a data availability statement. This statement should provide the following information, where applicable:

- Accession codes, unique identifiers, or web links for publicly available datasets
- A description of any restrictions on data availability
- For clinical datasets or third party data, please ensure that the statement adheres to our policy

Data availability: All sequencing data (fastq), variant calls (ancestry masked and unmasked VCFs), and metadata (anonymised individual IDs and locations) have been deposited in the Australian National Computational Infrastructure (NCI), Canberra under project identifier TE53. Access can be requested in writing to the NCIG Collection Access and Research Advisory Committee (CARAC), overseen by the Indigenous majority NCIG Board, by emailing jcsmr.ncig@anu.edu.au. Requests for data access for external research will be assessed in accordance with the NCIG Governance Framework available at https://ncig.anu.edu.au/files/NCIG-Governance-Framework.pdf. The data is available for general research use subject to meeting the requirements of the NCIG Governance Framework.
GRCh38.p13 - Human Genome assembly GRCh38.p13
EGAD00001001634 - Papuan Genomes: high depth (30x) whole genome sequence data - https://ega-archive.org/datasets/EGAD00001001634
PRJNA314367 - Genetic history of Melanesian individuals - https://www.ncbi.nlm.nih.gov/bioproject/?term=PRJNA314367
EGAD00010001326 - Papuan_Genotyping - https://ega-archive.org/studies/EGAS00001001587

Human research participants

Policy information about studies involving human research participants and Sex and Gender in Research.

Reporting on sex and gender	Approximately equal males and females were included.
Population characteristics	Geographic sampling locations were an important variable of this work. No phenotypic information was included. Variables such as age do not affect the results of this study.
Recruitment	The selection of communities was partly based on inclusion of diverse language groups, logistical access, and the presence of historical samples in the NCIG collection.<br>Within communities there was the possibility of non-random sampling with respect to genetics, i.e. including multiple samples from within a family. We addressed this by obtaining the largest sample size possible and excluding individuals based on genetic kinship estimates. Participants were recruited by volunteering, so we cannot exclude the possibility of some bias due to propensity to volunteer, but otherwise the sampling of individuals is random.
Ethics oversight	ANU ethics protocol 2015/065<br>University of Melbourne Ethics protocol 1852770<br>NCIG Governance Board oversight and approval

Note that full information on the approval of the study protocol must also be provided in the manuscript.

Field-specific reporting

Please select the one below that is the best fit for your research. If you are not sure, read the appropriate sections before making your selection.

☒ Life sciences ☐ Behavioural & social sciences ☐ Ecological, evolutionary & environmental sciences

For a reference copy of the document with all sections, see nature.com/documents/nr-reporting-summary-flat.pdf

Life sciences study design

All studies must disclose on these points even when the disclosure is negative.

Sample size	Sample size (which is clearly defined in the methods) was determined by the limitations of community engagement, but is the largest sample of Indigenous Australian genomes to date. The manuscript explores in detail the relationship between sample size and variant recovery.
Data exclusions	There are four levels of data exclusion.<br>1. The variant calls from a single sample were consistent with DNA cross-contamination, thus this sample was excluded for technical reasons.

2. 10 samples showed evidence of recent ancestry for both the Tiwi and a non-Tiwi Island population and were considered separately from the other Tiwi individuals.
3. Genomic regions of non-Indigenous ancestry were masked in most analyses. The decision to mask non-indigenous ancestry was pre-established and carried out using appropriate reference panels.
4. Exclusions due to kinship. We sought a sample of unrelated individuals so we used genetics to identify closely related individuals and excluded as many samples as required to give an unrelated sample. This is discussed clearly in the manuscript.
All four exclusions are clearly discussed and justified in the manuscript.

Replication	Sub-sampling and re-sampling were carried out where appropriate for the methods used, and this is noted in the text e.g. Figure 1 and Figure 5. Several individuals were sequenced twice to estimate variant call error rates.
Randomization	Participants were not allocated to experimental groups. This is essentially a descriptive study. Clearly geographic sampling location was the key grouping considered, but this is not randomization by the researchers. For some analyses we subsampled from the groups to ensure fair comparisons. In these cases the subsampling was random (with some conditions such as seeking to maintain samples with the greatest inferred Indigenous ancestry. In all case this is clearly explained in the text.
Blinding	This is not a randomized control trial and blinding is not necessary. That said, the researchers were blind to the identities of the participants and only knew their sampling location along with their genomic data. For a descriptive study, such as this, blinding is not necessary.

Reporting for specific materials, systems and methods

We require information from authors about some types of materials, experimental systems and methods used in many studies. Here, indicate whether each material, system or method listed is relevant to your study. If you are not sure if a list item applies to your research, read the appropriate section before selecting a response.

Materials & experimental systems

n/a	Involved in the study	
☒	☐	Antibodies
☒	☐	Eukaryotic cell lines
☒	☐	Palaeontology and archaeology
☒	☐	Animals and other organisms
☒	☐	Clinical data
☒	☐	Dual use research of concern

Methods

n/a	Involved in the study	
☒	☐	ChIP-seq
☒	☐	Flow cytometry
☒	☐	MRI-based neuroimaging
